# Feasibility of online group schema therapy: A preliminary study with therapists in training for future application in borderline personality disorder

**DOI:** 10.1016/j.invent.2025.100897

**Published:** 2025-12-05

**Authors:** Bram van der Boom, Tara Donker, Derek de Beurs, Arnout C. Smit, Lian van der Krieke, Pepijn Steures, Roel Pietersen, Marieke van Meeteren, Arnoud Arntz, Heleen Riper

**Affiliations:** a Department of Clinical, Neuro- and Developmental Psychology, Amsterdam Public Health Research Institute, Vrije Universiteit Amsterdam, Amsterdam, Netherlands; b Amsterdam Public Health Research Institute, Vrije Universiteit, Amsterdam, Netherlands; c Institute of Psychology, Department of Biological Psychology, Clinical Psychology and Psychotherapy, Albert Ludwigs-University of Freiburg, Freiburg im Breisgau, Baden-Württenberg, Germany; d Department of Clinical Psychology, University of Amsterdam, Amsterdam, Netherlands; e GGZ Drenthe, Hoogeveen, Netherlands; f University Center for Psychiatry, University Medical Center, University of Groningen, Netherlands; g Solutalks GGZ, Utrecht, Netherlands; h Pietersen Psychotherapie & Coaching, Hengelo, Netherlands; i KP Praktijk, Schematherapiecocon, Hengelo, Netherlands; j Amsterdam UMC, Location Vrije Universiteit Amsterdam, Department of Psychiatry, Amsterdam, Amsterdam Public Health Research Institute, Amsterdam, Netherlands

**Keywords:** Feasibility study, Schema therapy, eHealth, Group therapy, Non-patients, Personality disorder

## Abstract

**Background:**

Borderline personality disorder (BPD) is the most prevalent personality disorder and can significantly impair patients' functioning. Evidence-based treatments exist, but can be inaccessible due to various limitations. Internet-delivered treatment could potentially increase accessibility and scalability. Before investigating patients, we planned a pilot-test of video-conferencing group schema therapy (VC-GST) as self-therapy for therapists in training.

**Objective:**

This study aimed to assess the feasibility of VC-GST for therapists in training. We hypothesised VC-GST to be a feasible intervention.

**Methods:**

An uncontrolled pre-post-test feasibility study was conducted on a group of 24 therapists in training and analysed via one-sample and paired-samples *t*-tests. Feasibility was assessed through system usability, client satisfaction, group cohesion, working alliance, and drop-out rates. Furthermore, the effect of the VC-GST intervention on the participants' functioning, patterns, and personality traits was evaluated.

**Results:**

VC-GST showed high usability and strong client satisfaction, with marked improvement in group cohesion and working alliance, and a drop-out rate of 4 %. Additionally, participants showed an increase in adaptive and decrease in maladaptive schemas.

**Conclusions:**

This study demonstrates that VC-GST could be a feasible intervention for therapists in training, warranting further research into VC-GST for a clinical population of BPD patients.

## Highlights

• Feasibility study of video-conferencing schema therapy with therapists in training
• VC-GST led to improvements in ADP-IV personality traits, schemas, and coping modes.
• VC-GST is a feasible internet-delivered intervention for non-patient populations.
• Findings support further research into VC-GST for borderline personality disorder.

## 1 Background

### 1.1 Personality disorders

Personality disorders (PDs) form a substantial public health problem, as they affect around 8–12 % of the general adult population (Volkert et al., 2018; Winsper et al., 2020). PDs are recognized by maladaptive patterns of cognition, affect, and behaviour that are inflexible and enduring, resulting in impairment in social functioning and frequent distress (Volkert et al., 2018; Winsper et al., 2020). They are also known to have a high comorbidity with other psychiatric conditions and with alcohol misuse (Feenstra et al., 2012), and are associated with a low quality of life (Verheul et al., 1998). Importantly, PDs form a large economic burden, due to substantial use of health services as well as high absenteeism and productivity losses (Chiesa et al., 2002, Soeteman et al., 2008). Because PDs are chronic in nature, they were long thought to be untreatable. Since the 1990s, multiple evidence-based psychotherapies have demonstrated considerable success in treating PDs, particularly with borderline PD patients (BPD) (Torgersen, 2012). Given that BPD is the most prevalent PD (Torgersen, 2012), this study places focus upon this PD. BPD is characterised by impaired personal functioning, suicidal behaviour, therapeutic resistance and a high prevalence of both mental and physical comorbidities (Samuels, 2011; Gabbard, 2012). Suicidal behaviour remains prevalent worldwide, with notably higher suicide rates in non-European regions (Bertuccio et al., 2024). In the United States, suicide rates among men were the highest globally (15.5 per 100,000), increasing by 3.8 % per year between 2009 and 2020; among women, rates rose by 6.7 % annually from 2007 to 2017 before levelling off (Bertuccio et al., 2024). This underscores the importance of treating the PDs, particularly BPD, that are associated with suicidal behaviour. Treatments for BPD include schema therapy (ST) (Young et al., 2003), dialectic behavioural therapy (DBT) (Linehan et al., 2006), transference-focused psychotherapy (TFP) (Kernberg et al., 2008), and mentalization-based treatment (MBT) (Bateman and Fonagy, 2010). A meta-analysis by Oud et al. (2018) showed that these treatments (DBT, ST, MBT and TFP), exhibit a medium effect at post-treatment in reducing overall BPD severity (standardised mean difference (SMD) –0.59 [95 % CI: −0.90, −0.28]), when compared to treatment as usual (TAU) or community treatment by experts, based on a moderate quality evidence. These treatments, compared to waiting-list controls, reduced BPD symptoms in individuals as well as in group settings.

### 1.2 Schema therapy (ST)

In the last decades, ST has gained increasing popularity as a treatment for BPD (Sempértegui et al., 2012). It is an integrative psychotherapy that draws from various existing therapies, such as attachment theory, psychoanalytic object relations theory, cognitive behavioural therapy, and Gestalt therapy (Young et al., 2003). The underlying premise of ST is that maladaptive patterns, known as schemas, develop because of unmet needs during childhood. ST is among the most protocolized and researched interventions for BPDs, and is also administered in a group format (Arntz et al., 2022; Farrell et al., 2009; Oud et al., 2018).

Multiple studies have demonstrated the effectiveness of ST, primarily for adults, in both individual (Giesen-Bloo et al., 2006) and group therapy formats (Farrell et al., 2009). A multicentre RCT (*N* = 495) found that combined individual and group schema therapy was significantly more effective than optimal treatment as usual and predominantly group schema therapy in reducing BPD severity (Arntz et al., 2022). Group therapy offers several benefits when combined with individual therapy, including a supportive environment for practising new learned behaviours and interpersonal skills with peers (Arntz et al., 2022).

### 1.3 Limitations of face-to-face (F2F) therapy

While face-to-face (F2F) schema therapy for BPDs is evidence-based, there are several barriers that prevent its widespread implementation. For example, limited access to evidence-based psychotherapies is a common issue for patients in rural areas, with trained therapists mainly living in urban areas (Samuels, 2011). In addition, the therapies are typically time-consuming, often lasting between one and three years and requiring a significant level of intensity, resulting in long waitlists and high costs (Oud et al., 2018; Jacob and Arntz, 2013; Taylor et al., 2017; Perry et al., 1999; Biskin, 2015). To address some of these barriers, clinicians have explored the effects of shorter forms of these specialised psychotherapies. Despite limited research in this area, findings have been promising, with reduced frequency and duration of schema therapy producing comparable outcomes and drop-out rates to those in an earlier clinical trial (Nadort et al., 2009). Skewes et al. (2015) describes similar results in a feasibility study on short-term schema therapy for mixed personality disorders.

### 1.4 Internet-delivered therapy: a possible solution

One potential solution to addressing the accessibility and scalability of mental health treatment is internet-delivered therapy, which has demonstrated success in treating several common mental health disorders, such as anxiety disorders (Andersson et al., 2019), depression (Karyotaki et al., 2021), and alcohol use disorder (Riper et al., 2018; Ferreri et al., 2018). Various methods of delivering internet interventions in mental and behavioural health exist, the most developed and evaluated being internet-delivered interventions with guided email contact (Ebert et al., 2018). In addition, there are chat groups, mobile applications, video conferencing (VC) with a therapist and unguided internet-delivered interventions (Ebert et al., 2018; Cuijpers et al., 2017; Cuijpers and Riper, 2014). Either as a replacement or an addition to F2F therapy, internet-delivered interventions have the potential to increase access to mental health care and improve the effectiveness of treatment when used as an add-on (Cuijpers and Riper, 2014). Although there has been some research focusing on internet-delivered treatment for BPD with promising results, the research into these interventions is still in its infancy (Van der Boom et al., 2022). VC has potential advantages over F2F therapy: it helps minimize the impact of inadequate numbers and unequal distribution of appropriately trained therapists across the country, it may lower subsequent long wait times, reduce costs and minimize travel times (Basnet et al., 2014).

Until the COVID-19 pandemic started in 2020, VC was mainly used for professional consulting by general practitioners, psychiatrists and psychologists or for patient-therapist interactions in very remote areas such as the North of Scandinavia or Australia (Gaebel et al., 2020). However, the mandatory social distancing during the pandemic has led to the implementation of psychotherapy treatments delivered by VC on a large scale, making this current study even more relevant (De Beurs et al., 2022).

### 1.5 Internet-delivered video-based group ST (VC-GST)

Considering the effectiveness of group ST for patients with BPDs, and the possible advantages of internet-delivered therapy, we have developed a VC-Group schema therapy (VC-GST). The protocol for this therapy was based on, among others, a widely used protocol for short term schema-based cognitive behavioural group therapy in the Netherlands (Van Vreeswijk et al., 2006, Van Vreeswijk and Broersen, 2013; Van Vreeswijk et al., 2012; Skewes et al., 2015). This protocol was combined with experiential techniques from other existing schema therapy protocols and adapted for online use (Farrell et al., 2009). This was done by adding and adapting several typical F2F exercises, such as chair work, to an online format. Chair work refers to experiential interventions that utilise chairs, their positioning and movement to bring about change by enabling the therapist and their clients to have dialogues among various parts of the self (modes) (Kellogg and Young, 2006; Pugh et al., 2021).

Schema therapy (ST) was chosen over other evidence-based treatments due to our team's expertise, enabling us to design an effective online ST intervention. Additionally, while evidence-based treatments such as MBT and DBT are mainly used as a treatment for BPD, ST has also shown to be promising for other personality disorders and syndrome disorders such as depression and eating disorders (Bamelis et al., 2013; Bernstein et al., 2023; Joshua et al., 2022; Wibbelink et al., 2023).

Before testing this new delivery method of group ST on a BDP population, we chose to first offer VC-GST to healthy therapists in training, as part of a personal therapy process. Therapists in training are often expected to undergo personal therapy as part of their curriculum. This training therapy is thought to help them become more aware of their own personal functioning. Importantly, it allows them to experience what it is like to actually be in the position of a patient. Research into the effects of this training therapy is rare, but does suggest positive effects on their personal and professional development, as well as their levels of self-reflection and empathy (Van Dijk et al., 2022; Orlinsky et al., 2011).

This paper outlines the rationale behind the development of VC-GST and presents the results of a pilot study conducted with therapists in training. Testing the intervention on healthy therapists serves as a preliminary step to assess its feasibility. If proven feasible, the next phase would involve testing the intervention with BPD patients.

The primary aim of this study was to evaluate the feasibility of VC-GST for therapists in training, assessed through usability, client satisfaction, group cohesion, working alliance, and dropout rates. Following from this, our primary hypothesis was that VC-GST would be capable of fostering sufficient group cohesion—a key factor for effective group therapy—even in an online setting. A secondary aim was to explore the intervention's effects on participants' functioning, behavioural patterns, and personality traits. We therefore further hypothesise that this intervention would improve participants' overall functioning and personality traits.

## 2 Methods

### 2.1 Study design and procedure

A single-group uncontrolled pre-post-test feasibility study was conducted.

### 2.2 Participants

Participants were recruited between February 1 and April 30 in 2019, via the LinkedIn profile of the first author (BvdB)(see annex 1 for the full text).

In the recruitment period, 112 applications via email were received. Inclusion criteria required participants to be a group therapist- or healthcare/clinical psychologists in training (hereafter referred to as “therapists in training”), willing to take part in the intervention, having 1.5 h available time per week for ST sessions, having access to a desktop or laptop computer with webcam and adequate internet connection. Exclusion criteria were (−) psychotropic medication (unless they had been on stable dosage for the previous three months with no planned changes during the study period) or drugs; (−) therapists in training with self-harming or suicidal behaviour (as reported during screening); (−) or in need of primary treatment for another debilitating physical condition such as cancer. These exclusion criteria were assessed verbally during a screening call by the first author by means of yes/no questions.

Those who met the inclusion criteria and consented to participate were then invited by email to fill out a baseline questionnaire and subsequently the participants were assigned to one of the three videoconferencing (VC) groups based on their time availability, to start the intervention two weeks after the baseline measurement. Subsequently, the participants received instructions by email about how to participate, download and use Zoom. The study was approved by the Scientific and Ethical Review Board (VCWE) of VU Amsterdam (VCWE-2018-141 / Dec 8th 2018). Paper-pencil informed consent was obtained for all participants after the procedures had been fully explained.

### 2.3 Sample size

The aim of our study was to assess if our newly developed VC intervention could be delivered as intended and whether participants would engage and adhere to its components (Teresi et al., 2021). We therefore applied a convenience sample of *N* = 24, which may be deemed as sufficient for studies of this kind (Billingham et al., 2013). This number of participants enabled three groups consisting of seven to nine participants each, eight to nine being the standard size of GST (Arntz et al., 2022).

### 2.4 VC-Group ST intervention

An VC-GST protocol (see annex 2) was developed by combining aspects of F2F schema group therapy protocols and methods, that focused on cognitive and/or experiential techniques and adapting the techniques for online application (Van Vreeswijk et al., 2006, Van Vreeswijk and Broersen, 2013; Farrell et al., 2014; Skewes et al., 2015). The protocol consisted of 18 ST sessions in group online format with a duration of 1.5 h, and two group booster sessions of one hour, the first session one month after the initial 18 weeks, the second a subsequent two months later, thus 20 in total. The therapy sessions were given by three registered schema therapists who had a minimum of three years' experience with group therapy, being a psychiatrist (BVDB and PS) or licensed psychologists (RP and MVM). The participants were provided with an online workbook (see annex 3), outlining every session and containing homework for the next session. Aside from the 20 group therapy sessions via VC, participants received three individual online sessions with the group therapists to set and evaluate their individual goals. These individual online VC sessions took place at the start of the intervention (intake), and midway and the end of the treatment (evaluations). To locate underlying patterns and assess changes in these patterns, (prior to) each session the YSQ-2 and SMI were scored and evaluated, this being a standard part of schema therapy.

Furthermore, after eight sessions, the participants received a package by post with several items, of which some were used in therapy exercises and others served as transitional objects (see annex 4). For example, participants received a pebble that could be held in times of need, reminding patients of their part in this group. This use of transitional objects as a part of limited reparenting is common in different forms of schema therapy, such as the Farrell and Shaw method (Farrell et al., 2014).

Additional exercises were done to help form a therapeutic connection via VC, such as ‘joining hands’ through the screen, or doing the ‘wave’. Also, existing schema therapy exercises, such as chair work, were adapted to be used online. For example, we asked the participants to prepare for a chair technique by bringing additional chairs in their room before the session, asking them to sit on different chairs in front of the camera (individual chair work in the group).

Participants were not allowed to miss more than two sessions in total, or they would be excluded from the therapy, as they would miss too much of the therapy and the group process could be troubled by their infrequent attendance.

Each group session was accessed with a unique private individual code. The sessions were not recorded.

### 2.5 Measures

Measures were taken at baseline and nine months after baseline. Participants received the questionnaires via email and could either fill it in on their computer and email it back, or print it and send a paper version back to the first author.

After completing the group intervention, participants received a certificate of participation that could be used to obtain credits in their individual training program.

### 2.6 Primary outcome: feasibility

Different aspects of feasibility were assessed with the following instruments:

#### 2.6.1 Usability

The usability of the ZOOM platform was measured with the SUS, which is composed of 10 statements that are scored on a 5-point scale of strength of agreement. Composite scores for the SUS can range from 1 to 100 and were calculated upon the process originally devised by Brooke (1996, System Usability Scale (SUS) Score Calculator, https://stuart-cunningham.github.io/sus/). Higher scores indicate better usability; products that are at least passable have SUS scores above 68 (Sauro and Lewis, 2016; Lewis, 2018). Internal consistency of the SUS is high, (Cronbach's a = 0.91) (Bangor et al., 2009). A study by Mol et al. (2020) confirmed that the total sum score of the SUS appears to be a valid and interpretable measure to assess the usability of internet-based interventions when used by professionals in mental healthcare.

#### 2.6.2 Satisfaction with the intervention

Satisfaction with the intervention was assessed with the CSQ-8, an 8-item self-report measure. The 8-item self-report questionnaire scale response options are from 1 (lowest client satisfaction) to 4 (highest client satisfaction) with different anchors depending on the item; total score ranges from 8 to 32 (De Brey, 1983). If participants scored an average of >3 out of 4 on the Client Satisfaction Questionnaire 8 (CSQ-8) (De Brey, 1983), we assume that they were satisfied with the intervention. De Brey found a high internal consistency (Cronbach's α = 0.91), similar to the original English version (Cronbach's α = 0.93).

#### 2.6.3 Group therapeutic environment

The GCQ-S is a 12-item self-report measure that assesses individual group member's perceptions of the group's therapeutic environment. It consists of 12 items rated on a 7-point Likert scale indicating extent of agreement ranging from ‘not at all’ (0) to ‘extremely’ (6). The maximum score therefore is 72. The GCQ-S consists of 3 subscales, starting with the Engagement scale (range = 0–30). This describes constructive therapeutic work, including a positive working atmosphere, cognitive understanding, confrontation, group cohesion, and self-disclosure. The Conflict scale (range = 0–18) indicates interpersonal anger, distancing, distrust, and tension. And lastly the Avoidance scale (range = 0–24) includes ways members might avoid constructive involvement, such as avoiding issues between members, depending on the group leader, and engaging in high social monitoring (Johnson et al., 2006). In literature, primarily the 3 factor-derived subscales of the GCQ-S are being used to assess the group therapeutic environment and have high internal consistencies and thoroughly tested construct validity (Kivlighan Jr and Goldfine, 1991, Ryum et al., 2009, Tschuschke and Greene, 2002). The GCQ-S was administered three weeks into the intervention (after session 3) and at post-test 32 weeks after baseline assessment.

#### 2.6.4 Working alliance with the therapist

The CTS-1 (see annex 5) is a self-administered questionnaire that assesses the working alliance of the individual group member with the therapist. The CTS-1 assesses the participant's perceptions of the relationship with the group therapist that is most important to the participant. It contains 10 items rated on a 6-point Likert scale indicating the extent of agreement ranging from ‘not at all applicable’ (0) to ‘totally applicable’. For the CTS-1, no norm scores were available as the test has not been used extensively in research before.

### 2.7 Secondary outcomes: effects of VC-GST on therapists in training

Personal functioning was tested with the following instruments at baseline and post-test.

The ADP-IV is a self-report measure of the DSM-IV Axis II PDs (Schotte et al., 1998). The ADP-IV assesses for each DSM-IV criterion its typicality on a 7 point scale, as well as the accompanying distress and impairment, when typicality is rated >4, on a 3 point scale. We used the sum score of all items, representing general PD-pathology (Schotte and De Doncker, 2000).

The Young Schema Questionnaire 2 (YSQ-2) is a 205 item, self-administered questionnaire which assesses 16 subscales representing early maladaptive schemas (Rijkeboer, 2012). Lower average scores on the YSQ-2 indicate less psychopathology. As an outcome we used the average of all subscale scores. Internal consistency of the subscales of the YSQ-2 has proven to be high (Rijkeboer and Van Den Bergh, 2006).

The Schema Mode Inventory (SMI) is a 118-item self-administered questionnaire. It shows the frequency of schema modes the person reports to experience. There are 14 modes the person can score on (Young et al., 2007; Lobbestael et al., 2010). As an outcome we used the mean of the combined adaptive modes (happy child and healthy adult modes) and the mean of the combined maladaptive modes (all other modes).

The YSQ-2 and SMI were also used in the intake to set goals for the participants, and to evaluate the treatment half-way and at the end of the treatment as part of the treatment. Participants' YSQ-2 and SMI scores at baseline and end of treatment were used in the study to assess personal functioning for research purposes.

### 2.8 Statistical analysis

Descriptive statistics were used to report (socio)demographic variables and the primary outcome working alliance (CTS-1). Missing data was deleted listwise. A pre-post test design was used. The primary outcomes usability (SUS), client satisfaction (CSQ-8) were assessed at pos*t*-test, using one sample *t*-tests against the pre-set criterion values (testing that the mean rating was significantly above the criterion). The outcome group cohesion (GSQ-S) was tested using paired-samples t-test. Lastly, a binomial test was conducted to compare the expected completion rate of 0.75 (18/24) to the observed completion rate of the treatment.

To assess the secondary outcomes, paired-samples t-tests were conducted to compare the participants' total ADP-IV scores, total YSQ-2 scores and total SMI scores at pretest and post-test. Statistical analyses were performed using standard packages available in the R software language 2023 (R Core Team, 2023).

## 3 Results

### 3.1 Participants

**Fig. 1:**
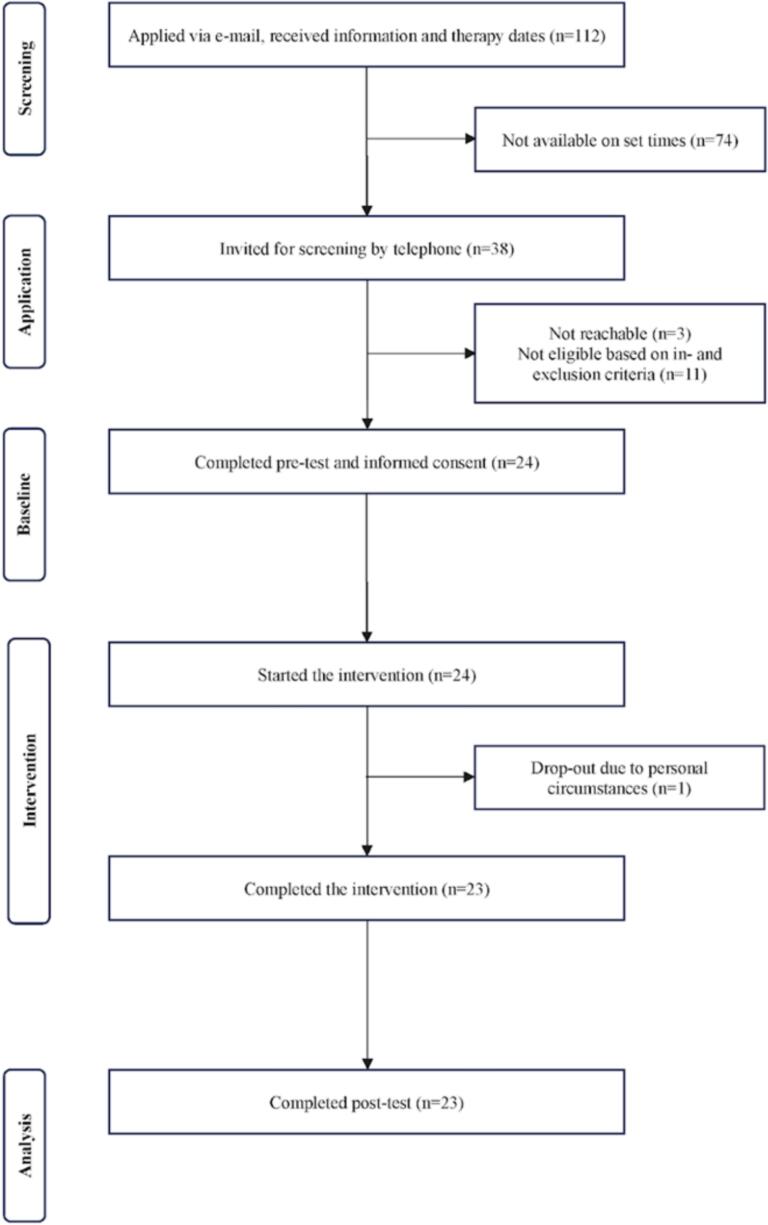
CONSORT flow diagram of study participants.

Fig. 1 shows the flow of the study participants. In total, 112 individuals applied. Out of those, 74 individuals were excluded due to not being available on the set dates. Subsequently, screening by telephone was used to ensure participants fit the in- and exclusion criteria. In total, 11 participants had to be excluded after the screening conversation due to fitting the exclusion criteria. Out of these, 7 participants were excluded due to being on psychotropic medication and having planned dosage changes during the study period. Additionally, 3 participants were engaging in self-harming behaviour, meaning they were not able to be included in the study. Finally, one participant was unable to be included due to being in a state of crisis. Furthermore, 3 individuals could not be reached and were therefore not included, leading to 24 eligible participants. These participants were provided with informed consent and completed a pretest at baseline, followed by the start of the intervention. During the intervention, one participant had to drop out of treatment due to personal circumstances, leading to a total of 23 participants to complete the intervention.

The study included a sample of 24 therapists in training, being 20 psychologists, one psychotherapist, one developmental psychologist, one remedial educationalist, and one occupational therapist. Three of the participants participated in the study to fulfil (a part of) their mandatory personal therapy for their training. Due to changes in the availability of group members, we ended up with three different sized groups. The sociodemographic characteristics of the sample are presented in Table 1. As can be seen in Table 1, there were more female than male participants. The age range of the participants of the study was 26 to 56 years old.

**Table 1:** Sociodemographic characteristics of the sample at baseline.

Group	Sex male (%)	Age years range (M, mean)
Total (N = 24)	5 (21 %)	26–56 (38.6)
Group 1 (*n* = 9)	2 (22 %)	27–44 (35.7)
Group 2 (*n* = 7)	2 (29 %)	27–56 (42.1)
Group 3 (*n* = 8)	1 (13 %)	26–50 (38.8)

### 3.2 Primary outcome: feasibility

#### 3.2.1 Usability (SUS)

At post-test, participants scored an average of 75.65 (SD = 13.92; range 37–95) on the SUS regarding the usability of the ZOOM platform, which is significantly above the preset criterium (average) of 68 (t(23) = 2.64, *p* < 0.02) and can be translated into the system evaluation adjective ‘Good’ (Bangor et al., 2009). Of the 23 participants, 17 (74 %) scored usability >68.

#### 3.2.2 Client satisfaction (CSQ-8)

Participants rated satisfaction with an average score of 3.25 (SD = 0.48; range 2.50–3.88) on the CSQ-8, on a scale from 1 to 4, which is significantly above the preset criterion of 3.0 (t(23) = 2.50, p < 0.02), meaning they were satisfied.

#### 3.2.3 Group cohesion and working alliance (GCQ-S and CTS-1)

On the GCQ-S, there was a significant increase between the total score at three weeks into the intervention (T0) and at post-test (T1). This means the experienced group cohesion of participants increased significantly over time. More specifically, there was an statistically significant increase on the subscale “Engagement” as well as a decrease on the subscale “Conflict” between the scores at T0 and T1. The decrease on the subscale of “Avoidance” from pre- to post-test was statistically insignificant. The results are shown in Table 2.

**Table 2:** Descriptives and tests of changes of the GCQ-S.

	T0 mean (N = 24)	T1 mean (*N* = 23)	D.F.	t-Value	*p*-Value
**Engagement**	21.18 (SD = 2.54)	24.95 (SD = 2.57)	21	6.67	< 0.001
**Avoidance**	10.36 (SD = 2.50)	9.68 (SD = 2.78)	21	−0.86	0.408
**Conflict**	7.05 (SD = 3.56)	4.95 (SD = 2.38)	20	−2.28	0.034
**Total**	45.76 (SD = 6.04)	52.32 (SD = 5.38)	20	4.29	< 0.001

At post-test participants scored an average of 4.37 (SD = 0.50) on the CTS-1, on a scale from 1 to 6, meaning the therapeutic alliance was scored relatively high (see Table 3).

**Table 3:** Items and scores of the CTS-1.

Item	Mean	Range
I trust him/her (the therapist)	5.13	4–6
I wish he/she was more open about his/her feelings	4.46	2–6
I would like to make friends with him/her outside the group	2.83	1–5
I respect him/her	5.30	4–6
I wish he/she would pay more attention to me	4.46	3–6
I know him/her	2.5	1–5
I like him/her	4.75	4–6
I wish he/she would be more active	4.63	2–6
I am like him/her (I share some interests with him/her)	3.33	1–6

### 3.3 Drop-out rate

One participant dropped out of the treatment after 10 weeks for work-related reasons, giving a completion rate of 96 %. A binomial test yielded a chance of 0.009 that with an expected proportion of completers of 0.75 (i.e., 18/24), the completion rate would be 23 or higher. Thus, the observed completion rate was significantly higher than the preset criterion.

### 3.4 Secondary outcomes: effects of VC-GST on therapists in training

Table 4 shows the results for the effects of the VC-GST on the personal functioning of therapists in training. At baseline, the group scored below average on the ADP-IV compared to scores from a psychiatric sample (Schotte et al., 2004). This indicates that, although the healthy therapists-in-training exhibited some traits associated with personality disorders, their symptoms were not comparable to those of psychiatric patients. There was a significant medium decrease (*p* = 0.003) between the ADP-IV scores for general PD-pathology between pretest and post-test. At the end of the treatment, on average, the participants scored lower on traits associated with having a PD than they did at the beginning, indicating that their maladaptive patterns had become less rigid and active.

**Table 4:** Descriptives and tests of changes of the ADP-IV, YSQ-2, and SMI scores.

	T0 mean (*N* = 24)	T1 mean (*N* = 23)	D.F.	t-value	p-value
**ADP-IV**	139.79 (SD = 35.15)	120.89 (SD = 26.49)	21	−3.40	0.003
**YSQ-2**	1.85 (SD = 0.38)	1.65 (SD = 0.40)	22	−3.98	< 0.001
**Adaptive modes**	71.3 % (SD = 7.75 %)	76.1 % (SD = 11.5 %)	20	3.26	0.004
**Maladaptive modes**	45.76 (SD = 6.04)	17.1 (SD = 6.25)	20	5.20	< 0.001

As can be seen in Table 4, there was a significant decrease between the YSQ-2 scores between pretest and post-test. This meant that at the end of treatment on average the participants reported less active negative schemas at post-test compared to pretest, which is associated with better functioning.

Lastly, a medium significant increase in adaptive modes was found from pretest to post-test; a significant decrease in maladaptive modes was found from pretest to post-test (Table 4). This meant that at the end of treatment, on average, the participants spent less time in maladaptive modes of functioning at post-test compared to pretest.

## 4 Discussion

The findings presented in this paper indicated high usability and satisfaction ratings, with a low dropout rate (one participant). Group cohesion ratings increased significantly throughout the intervention. Notably, participant satisfaction in this non-patient cohort was comparable to that found in other studies, such as Van Schaik et al. (2022), who investigated a blended interpersonal psychotherapy (IPT) for major depressive disorder, and by Weidle et al. (2024), who explored cognitive-behavioural therapy (CBT) via video conferencing for paediatric obsessive-compulsive disorder (OCD).

The study observed notable changes in group cohesion over time, with an increase in engagement and a decrease in conflict (GCQ-S). Final scores on the subscales in this study were generally in line with those found in other group therapy studies and showed comparable levels of group cohesion, despite those studies being face-to-face interventions (e.g., Kanas et al., 1994; Kanas and Cox, 1998). However, the conflict score in this study was higher (M = 4.95) compared to previous findings (M = 1.10 and M = 1.50), along with engagement being significantly higher (M = 24.95 vs. M = 17.29 and M = 16.23). These differences highlight the possible variations in group dynamics across studies. The decrease on the subscale of avoidance was not significant, which aligns with ongoing discussions in the literature regarding the psychometric properties and factor structure of this subscale (Hurley and Brooks, 1987; Hurley and Brooks, 1988; Johnson et al., 2005; Ryum et al., 2009).

Group cohesion is a critical factor in the effectiveness of group therapy, as it fosters a sense of belonging, emotional support, and trust among participants. Strong group cohesion has been linked to better therapeutic outcomes, including increased engagement in sessions, greater willingness to share personal experiences, and improved adherence to therapeutic tasks. It can also enhance resilience against dropout and facilitate interpersonal learning, which is particularly relevant in schema therapy, where exploring relational patterns is a core component. In this study, a statistically significant increase was found for the total score of the participants on the GCQs, indicating a strong group cohesion over time, showing that group cohesion can be achieved via VC group schema therapy interventions. Lastly, the working alliance was found to be relatively high, as seen from the scores on the CTS-1. In conclusion, these results support our first hypothesis, which signals that VC-GST can foster sufficient group cohesion and is a feasible treatment for therapists in training. In this study, we also hypothesised that participants would show improvements in functioning as measured from pre- to post-test. The findings show that participants on average scored lower on traits that are typically associated with having a PD than before treatment start, indicating that maladaptive patterns had become less rigid. Lastly, participants reported less negative schemas, showed increases in adaptive modes, and had decreases in maladaptive modes at the end of treatment. Therefore, the findings of this study are consistent with the second hypothesis, for participants showed significant improvements in personal functioning, as measured by changes in personality traits, schemas, and modes, but causal inference is limited due to the lack of a control group.

From the findings of this study, a number of practical implications may be drawn. To our knowledge, this is the first study to investigate a fully VC group schema therapy. While previous research has focused on guided or blended internet interventions (Van der Boom et al., 2022), our findings indicate that further research into the feasibility of VC-GST for BPD patients is justified. This signals that the key components of a group schema therapy intervention, such as high client satisfaction and usability of the intervention, can be effectively adapted to an online VC-GST treatment method. Additionally, as VC-GST is a form of innovative internet-delivered treatment, it would provide scalability and accessibility of mental health treatment. In conclusion, these results support our suggestion that VC-GST can be feasible for therapists in training.

### 4.1 Strengths and limitations

A number of notable strengths can be identified for this study. One of these is that this study stands out as one of the few to investigate the effect of training therapy on a group of healthy psychologists. Given that the research on training therapy for psychologists is scarce (Orlinsky et al., 2011), this paper contributes a valuable addition to the field.

Another strength is that this study found preliminary evidence for the effect of an online video conferencing group schema therapy intervention. The literary review that has been conducted (Van der Boom et al., 2022), combined with the current proof of concept study provides initial evidence that BPD patients can derive satisfaction and benefits from therapy delivered via video conferencing. An online video conferencing group schema therapy intervention was created and showed preliminary statistically significant results, indicating that this therapy might be feasible for a clinical BPD population. A next step would then be to conduct a randomised controlled trial to further explore the effects of VC-GST for BPD patients.

Furthermore, several limitations can be identified for this study. First, the use of a nonclinical convenience sample, consisting of self-referred and volunteering therapists in training, may have influenced the results. Participants' awareness of the study's aims might have led to conscious or unconscious attempts to provide favorable responses, both at baseline and post-test. Additionally, the potential developer (BvdB) allegiance bias could have influenced the results in terms of effect and acceptance. Generalisability of these results to the broader group of therapists in training is therefore limited.

Also, while participants demonstrated significant improvements in functioning, as measured by changes in personality traits, schemas, and modes from pre- to post-test, caution is warranted in extrapolating these findings to clinical BPD populations. BPD is associated with substantial challenges, including difficulties in social interactions, therapeutic resistance, and susceptibility to crises and dissociation (Samuels, 2011; Gabbard, 2012). Delivering VC to individuals with BPD may introduce additional barriers, such as lack of engagement in a virtual setting, privacy concerns at home, feelings of unsafety, poor internet connectivity, and limited opportunities for therapists to intervene during crises (Paulik et al., 2021). These factors should be considered when interpreting the findings and assessing the feasibility of VC in clinical practice, although preliminary research suggests that BPD patients can derive satisfaction and benefits from therapy delivered via VC (Van der Boom et al., 2022).

Additionally, the therapy sessions were not recorded, which hindered the ability to perform observational analyses of protocol fidelity by the therapists and participant adherence, group cohesion, therapist-participant interactions, or adherence to the schema therapy model.

Furthermore, as this study primarily examined feasibility and involved a relatively small sample size, exploring subgroup analyses based on demographic characteristics such as gender and age was beyond its scope. However, such factors are relevant and could be examined in future, larger-scale trials of VC-GST interventions to determine whether demographic variables influence outcomes or treatment engagement. Additionally, no claims could be made about the long-term effects of VC-GST, as no longitudinal follow-up was conducted.

Moreover, the CTS-1, a measure for the working alliance with the therapist that was used in this study, has not been extensively validated and has limited use in prior research, which complicates the interpretation and reliability of its results.

Lastly, the virtual format of the intervention introduces unique challenges, such as potential variability in participants' home environments, internet connectivity, and privacy, which could have impacted engagement and outcomes. These contextual factors were not systematically measured or controlled in this study.

## 5 Conclusion

This paper presents the rationale for an online group schema therapy (VC-GST) for borderline personality disorder (BPD) and the results of an uncontrolled pilot study involving therapists in training. This proof-of-concept study demonstrated promising results with regards to feasibility, showing a good usability, high client satisfaction, a marked increase in group cohesion and working alliance, and a low drop-out rate. These findings indicate that the core components of schema therapy may also be transferrable to an online format, which signals that in the future, internet-delivered treatments such as VC-GST, may be implemented and could potentially effectively enhance the accessibility and scalability of therapy treatment. Furthermore, this research contributes a valuable addition to the scarce literature on training therapy for therapists, and contributes to the growing body of literature on the internet-delivered treatments. Additionally, these findings provide a preliminary support for the future adaptation of the VC-GST intervention in a clinical BPD population, although caution must be exerted when generalising these results to a clinical sample, as challenges such as therapeutic intervention during crises, privacy concerns at home, and lack of engagement, may plausibly occur in virtual settings. Although the current study was conducted on a relatively small sample of therapists in training and cannot be immediately extrapolated to a clinical population of BPD patients, the findings of this proof-of-concept study show preliminary findings that BPD patients may potentially derive satisfaction and benefits from treatment delivered via VC. These results are therefore promising for further research into VC-GST as a potential intervention and would warrant a feasibility study for VC-GST for a clinical population of BPD patients. A next step would then be to conduct a RCT to further explore the potential of a VC-GST intervention for patients with BPD.

The following are the supplementary data related to this article.

Annex 1 Recruitment participants. Annex 1

Annex 2 VC-GST Therapist Protocol. Annex 2

Annex 3 Patient workbook. Annex 3

Annex 4 Document transitional objects. Annex 4

Annex 5 CTS-1 Questionnaire. Annex 5

## Special thanks

We thank Lenneke Bergboer and Anne Lagemaat for their excellent support and enthusiasm, and the therapists in training for their participation in the study.

## Funding

This study has not received any specific grant from funding agencies in the public, commercial, or non-profit sectors.

## Declaration of competing interest

The authors declare that they have no known competing financial interests or personal relationships that could have appeared to influence the work reported in this paper.

## References

[bb0005] Andersson G., Carlbring P., Titov N., Lindefors N. (2019). Internet interventions for adults with anxiety and mood disorders: a narrative umbrella review of recent meta-analyses. Can. J. Psychiatr..

[bb0020] Arntz A., Jacob G.A., Lee C.W., Brand-de Wilde O.M., Fassbinder E., Harper R.P., Lavender A., Lockwood G., Malogiannis I.A., Ruths F.A., Schweiger U., Shaw I.A., Zarbock G., Farrell J.M. (2022). Effectiveness of predominantly group schema therapy and combined individual and group schema therapy for borderline personality disorder: a randomized clinical trial. JAMA Psychiatry.

[bb0025] Bamelis L.L., Evers S.M., Spinhoven P., Arntz A. (2013). Results of a multicenter randomized controlled trial of the clinical effectiveness of schema therapy for personality disorders. Am. J. Psychiatry.

[bb0030] Bangor A., Kortum P., Miller J. (2009). Determining what individual SUS scores mean: adding an adjective rating scale. J. Usability Stud..

[bb0035] Basnet S., Tamminen M., Lahti T. (2014). The feasibility of ehealth in mental health care. J Addict Res Ther.

[bb0040] Bateman A., Fonagy P. (2010). Mentalization based treatment for borderline personality disorder. World Psychiatry.

[bb0045] Bernstein D.P., Keulen-de Vos M., Clercx M., De Vogel V., Kersten G.C., Lancel M., Arntz A. (2023). Schema therapy for violent PD offenders: a randomized clinical trial. Psychol. Med..

[bb0050] Bertuccio P., Amerio A., Grande E., La Vecchia C., Costanza A., Aguglia A., Berardelli I., Serafini G., Amore M., Pompili M., Odone A. (2024). Global trends in youth suicide from 1990 to 2020: an analysis of data from the WHO mortality database. EClinicalMedicine.

[bb0055] Billingham S.A., Whitehead A.L., Julious S.A. (2013). An audit of sample sizes for pilot and feasibility trials being undertaken in the United Kingdom registered in the United Kingdom Clinical Research Network database. BMC Med. Res. Methodol..

[bb0060] Biskin R.S. (2015). The lifetime course of borderline personality disorder. Can. J. Psychiatr..

[bb0065] Brooke John, Jordan P.W., Thomas B., Weerdmeester B.A., McClelland I.L. (1996). Usability Evaluation in Industry.

[bb0070] Chiesa M., Fonagy P., Holmes J., Drahorad C., Harrison-Hall A. (2002). Health service use costs by personality disorder following specialist and nonspecialist treatment: a comparative study. J. Personal. Disord..

[bb5005] Cuijpers P., Kleiboer A., Karyotaki E., Riper H. (2017). Internet and mobile interventions for depression: opportunities and challenges. Depress. Anxiety.

[bb5000] Cuijpers P., Riper H. (2014). Internet interventions for depressive disorders: an overview. Rev. Psicopatol. Psicol. Clín..

[bb0075] De Beurs E., Blankers M., Peen J., Rademacher C., Podgorski A., Dekker J. (2022). Impact of COVID-19 social distancing measures on routine mental health care provision and treatment outcome for common mental disorders in the Netherlands. Clin. Psychol. Psychother..

[bb0080] De Brey H. (1983). A cross-national validation of the Client Satisfaction Questionnaire: the Dutch experience. Eval. Program Plann..

[bb0085] Ebert D.D., Van Daele T., Nordgreen T., Karekla M., Compare A., Zarbo C., Brugnera A., Øverland S., Trebbi G., Jensen K.L., Kaehlke F., Baumeister H., Taylor J. (2018). Internet- and mobile-based psychological interventions: applications, efficacy, and potential for improving mental health: A report of the EFPA E-Health Taskforce. Eur. Psychol..

[bb0090] Farrell J.M., Shaw I.A., Webber M.A. (2009). A schema-focused approach to group psychotherapy for outpatients with borderline personality disorder: a randomized controlled trial. J. Behav. Ther. Exp. Psychiatry.

[bb0095] Farrell J.M., Reiss N., Shaw I.A. (2014).

[bb0100] Feenstra D.J., Hutsebaut J., Laurenssen E.M.P., Verheul R., Busschbach J.J., Soeteman D.I. (2012). The burden of disease among adolescents with personality pathology: quality of life and costs. J. Personal. Disord..

[bb0105] Ferreri F., Bourla A., Mouchabac S., Karila L. (2018). e-Addictology: an overview of new technologies for assessing and intervening in addictive behaviors. Front. Psychol..

[bb0110] Gabbard G.O., Nemeroff C.B. (2012). Management of Treatment-resistant Major Psychiatric Disorders.

[bb0115] Gaebel W., Trost N., Diekmann S., Lukies R., Zielasek J. (March 2020). Transnational Policy for e-Mental Health. https://www.nweurope.eu/media/10450/emen__transnational-policy-for-e-mental-health_guidance-document_3-2020.pdf.

[bb0120] Giesen-Bloo J., van Dyck R., Spinhoven P., van Tilburg W., Dirksen C., van Asselt T., Kremers I., Nadort M., Arntz A. (2006). Outpatient psychotherapy for borderline personality disorder: randomized trial of schema-focused therapy vs transference-focused psychotherapy. Arch. Gen. Psychiatry.

[bb0125] Hurley J.R., Brooks L.J. (1987). Brief reports group climate’s principal dimension: affliation. Int. J. Group Psychother..

[bb5050] Hurley J.R., Brooks L.A. (1988). Primacy of affiliativeness in ratings of group climate. Psychol. Rep..

[bb0130] Jacob G.A., Arntz A. (2013). Schema therapy for personality disorders: a review. Int. J. Cogn. Ther..

[bb0135] Johnson J.E., Burlingame G.M., Olsen J., Davies D.R., Gleave R.L. (2005). Group climate, cohesion, alliance, and empathy in group psychotherapy: multilevel structural equation models. J. Couns. Psychol..

[bb0140] Johnson J.E., Pulsipher D., Ferrin S.L. (2006). Measuring group processes: a comparison of the GCQ and CCI. Group Dyn. Theory Res. Pract., [s. l.].

[bb0145] Joshua P.R., Lewis V., Kelty A.F., Boer D.P. (2022). Is schema therapy effective for adults with eating disorders? A systematic review into the evidence. Cogn. Behav. Ther..

[bb5040] Kanas N., Cox P. (1998). Process and content in a therapy group for bipolar outpatients. Group.

[bb5030] Kanas N., Schoenfeld F., Marmar C.R., Weiss D.S., Koller P. (1994). Process and content in a long-term PTSD therapy group for Vietnam veterans. Group.

[bb0150] Karyotaki E., Efthimiou O., Miguel C., Bermpohl F.M.G., Furukawa T.A., Cuijpers P., Riper H., Patel V., Mira A., Gemmil A.W., Yeung A.S., Lange A., Williams A.D., Mackinnon A., Geraedts A., van Straten A., Meyer B., Björkelund C., Knaevelsrud C., Forsell Y., Individual Patient Data Meta-Analyses for Depression (IPDMA-DE) Collaboration (2021). Internet-based cognitive behavioral therapy for depression: a systematic review and individual patient data network meta-analysis. JAMA Psychiatr..

[bb0155] Kellogg S.H., Young J.E. (2006). Schema therapy for borderline personality disorder. J. Clin. Psychol..

[bb0160] Kernberg O.F., Yeomans F.E., Clarkin J.F., Levy K.N. (2008). Transference focused psychotherapy: overview and update. Int. J. Psychoanal..

[bb0165] Kivlighan D.M., Goldfine D.C. (1991). Endorsement of therapeutic factors as a function of stage of group development and participant interpersonal attitudes. J. Couns. Psychol..

[bb0170] Lewis J.R. (2018). The system usability scale: past, present, and future. Int. J. Hum.-Comput. Interact..

[bb0175] Linehan M.M., Comtois K.A., Murray A.M., Brown M.Z., Gallop R.J., Heard H.L., Korslund K.E., Tutek D.A., Reynolds S.K., Lindenboim N. (2006). Two-year randomized controlled trial and follow-up of dialectical behavior therapy vs therapy by experts for suicidal behaviors and borderline personality disorder. Arch. Gen. Psychiatry.

[bb0180] Lobbestael J., van Vreeswijk M., Spinhoven P., Schouten E., Arntz A. (2010). Reliability and validity of the short Schema Mode Inventory (SMI). Behav. Cogn. Psychother..

[bb0185] Mol M., van Schaik A., Dozeman E., Ruwaard J., Vis C., Ebert D.D., Etzelmueller A., Mathiasen K., Moles B., Mora T., Pedersen C.D., Skjøth M.M., Peleteiro Pensado L., Piera-Jimenez J., Gokcay D., Ünlü Ince B., Russi A., Sacco Y., Zanalda E., Fullaondo Zabala A., Riper H., Smit J.H. (2020). Dimensionality of the system usability scale among professionals using internet-based interventions for depression: a confirmatory factor analysis. BMC Psychiatry.

[bb0190] Nadort M., Van Dyck R., Smit J.H., Giesen-Bloo J., Eikelenboom M., Wensing M., Spinhoven P., Dirksen C., Bleecke J., Van Milligen B., Van Vreeswijk M., Arntz A. (2009). Three preparatory studies for promoting implementation of outpatient schema therapy for borderline personality disorder in general mental health care. Behav. Res. Ther..

[bb0195] Orlinsky D.E., Schofield M.J., Schroder T., Kazantzis N. (2011). Utilization of personal therapy by psychotherapists: a practice-friendly review and a new study. J. Clin. Psychol..

[bb0200] Oud M., Arntz A., Hermens M.L., Verhoef R., Kendall T. (2018). Specialized psychotherapies for adults with borderline personality disorder: a systematic review and meta-analysis. Aust. N. Z. J. Psychiatry.

[bb5055] Paulik G., Maloney G., Arntz A., Bachrach N., Koppeschaar A., McEvoy P. (2021). Delivering imagery rescripting via telehealth: clinical concerns, benefits, and recommendations. Curr. Psychiatry Rep..

[bb0205] Perry J.C., Banon E., Ianni F. (1999). Effectiveness of psychotherapy for personality disorders. Am. J. Psychiatry.

[bb0210] Pugh M., Bell T., Dixon A. (2021). Delivering tele-chairwork: a qualitative survey of expert therapists. Psychother. Res..

[bb0215] R Core Team (2023). https://www.r-project.org/foundation/.

[bb0220] Rijkeboer M., van Vreeswijk M., Broersen J., Nadort M. (2012). The Wiley-Blackwell Handbook of Schema Therapy: Theory, Research, and Practice.

[bb5060] Rijkeboer M.M., Van Den Bergh H. (2006). Multiple group confirmatory factor analysis of the young schema-questionnaire in a Dutch clinical versus non-clinical population. Cogn. Ther. Res..

[bb0225] Riper H., Hoogendoorn A., Cuijpers P., Karyotaki E., Boumparis N., Mira A., Andersson G., Berman A.H., Bertholet N., Bischof G., Blankers M., Boon B., Boß L., Brendryen H., Cunningham J., Ebert D., Hansen A., Hester R., Khadjesari Z., Kramer J., Murray E., Postel M., Schulz D., Sinadinovic K., Suffoletto B., Sundström C., de Vries H., Wallace P., Wiers R.W., Smit J.H. (2018). Effectiveness and treatment moderators of internet interventions for adult problem drinking: an individual patient data meta-analysis of 19 randomised controlled trials. PLOS Med..

[bb0230] Ryum T., Hagen R., Nordahl H.M., Vogel P.A., Stiles T.C. (2009). Perceived group climate as a predictor of long-term outcome in a randomized controlled trial of cognitive-Behavioural group therapy for patients with comorbid psychiatric disorders. Behav. Cogn. Psychother..

[bb0235] Samuels J. (2011). Personality disorders: epidemiology and public health issues. Int. Rev. Psychiatry.

[bb0240] Sauro J., Lewis J.R. (2016).

[bb0245] Schotte C.K.W., De Doncker D. (2000). De adp-iv: een vragenlijst voor een therapeutisch georiënteerde diagnostiek van persoonlijkheidsstoornissen. Psychopraxis.

[bb0250] Schotte C.K.W., De Doncker D., Vankerckhoven C., Vertommen H., Cosyns P. (1998). Self-report assessment of the DSM-IV personality disorders. Measurement of trait and distress characteristics: the ADP-IV. Psychol. Med..

[bb0255] Schotte C.K.W., De Doncker D.a.M., Dmitruk D., Van Mulders I., D’Haenen H., Cosyns P. (2004). The ADP-IV questionnaire: differential validity and concordance with the semi-structured interview. J. Personal. Disord..

[bb5065] Sempértegui G.A., Karreman A., Arntz A., Bekker M.H. (2012). Schema therapy for borderline personality disorder: a comprehensive review of its empirical foundations, effectiveness and implementation possibilities. Clin. Psychol. Rev..

[bb0260] Skewes S.A., Samson R.A., Simpson S.G., van Vreeswijk M. (2015). Short-term group schema therapy for mixed personality disorders: a pilot study. Front. Psychol..

[bb0265] Soeteman D.I., Hakkaart-Van Roijen L., Verheul R., Busschbach J.J. (2008). The economic burden of personality disorders in mental health care. J. Clin. Psychiatry.

[bb0270] Taylor C.D., Bee P., Haddock G. (2017). Does schema therapy change schemas and symptoms? A systematic review across mental health disorders. Psychol. Psychother. Theory Res. Pract..

[bb0275] Teresi J.A., Yu X., Stewart A.L., Hays R.D. (2021). Guidelines for designing and evaluating feasibility pilot studies. Med. Care.

[bb0280] Torgersen S., Widiger T.A. (2012). The Oxford Handbook of Personality Disorders.

[bb0285] Tschuschke V., Greene L.R. (2002). Group therapists’ training: what predicts learning?. Int. J. Group Psychother..

[bb5010] Van der Boom B., Boumparis N., Donker T., De Beurs D., Arntz A., Riper H. (2022). Internet-delivered interventions for personality disorders – a scoping review. Internet Interv..

[bb0290] Van Dijk J., Bartak A., Wijts P. (2022). Leertherapie: practise what you preach. Tijdschr. Psychother..

[bb5025] Van Schaik, Schotanus A.Y., Dozeman E., Huibers M.J.H., Cuijpers P., Donker T. (2022). Pilot study of blended-format interpersonal psychotherapy for major depressive disorder. Am. J. Psychother..

[bb0295] Van Vreeswijk, Broersen J. (2006). Schemagerichte therapie in groepen: handleiding voor therapeuten.

[bb0300] Van Vreeswijk M.F., Broersen J. (2013).

[bb5020] Van Vreeswijk, Spinhoven P., Eurelings‐Bontekoe E.H.M., Broersen J. (2012). Changes in symptom severity, schemas and modes in heterogeneous psychiatric patient groups following short‐term schema Cognitive–Behavioural group therapy: a naturalistic pre‐treatment and post‐treatment design in an outpatient clinic. Clin. Psychol. Psychother..

[bb0315] Verheul R., Ball S.A., Van den Brink W., Kranzler H.R., Rounsaville B.J. (1998). Dual Diagnosis and Treatment: Substance Abuse and Comorbid Medical and Psychiatric Disorders.

[bb0320] Volkert J., Gablonski T.C., Rabung S. (2018). Prevalence of personality disorders in the general adult population in Western countries: systematic review and meta-analysis. Br. J. Psychiatry J. Ment. Sci..

[bb5080] Weidle B., Babiano-Espinosa L., Skokauskas N., Wolters L.H., Henriksen M., Arntzen J., Skare A., Ivarsson T., Groff T., Skarphedinsson G. (2024). Online CBT versus standard CBT for pediatric obsessive-compulsive disorder. Child Psychiatry Hum. Dev..

[bb0325] Wibbelink C.J.M., Venhuizen A.S.S.M., Grasman R.P.P.P., Bachrach N., van den Hengel C., Hudepohl S., Kunst L., de Lange H., Louter M.A., Matthijssen S.J.M.A., Schaling A., Walhout S., Wichers K.(R.), Arntz A. (2023). Group schema therapy for cluster-C personality disorders: a multicentre open pilot study. Clin. Psychol. Psychother..

[bb0330] Winsper C., Bilgin A., Thompson A., Marwaha S., Chanen A.M., Singh S.P., Wang A., Furtado V. (2020). The prevalence of personality disorders in the community: a global systematic review and meta-analysis. Br. J. Psychiatry J. Ment. Sci..

[bb0335] Young J.E., Klosko J.S., Weishaar M.E. (2003).

[bb0340] Young J.E., Arntz A., Atkinson T., Lobbestael J., Weishaar M.E., Van Vreeswijk M.F., Klokman J. (2007).

