# Supplementary material for: Feasibility of online group schema therapy: A preliminary study with therapists in training for future application in borderline personality disorder
Source: Internet Interv. 2025 Dec 5;43:100897. doi: 10.1016/j.invent.2025.100897 (PMC12768866; doi:10.1016/j.invent.2025.100897)
Supplement: Annex 1 — Recruitment participants. [file mmc1.docx]

**Annex 1- Recruitment participants**


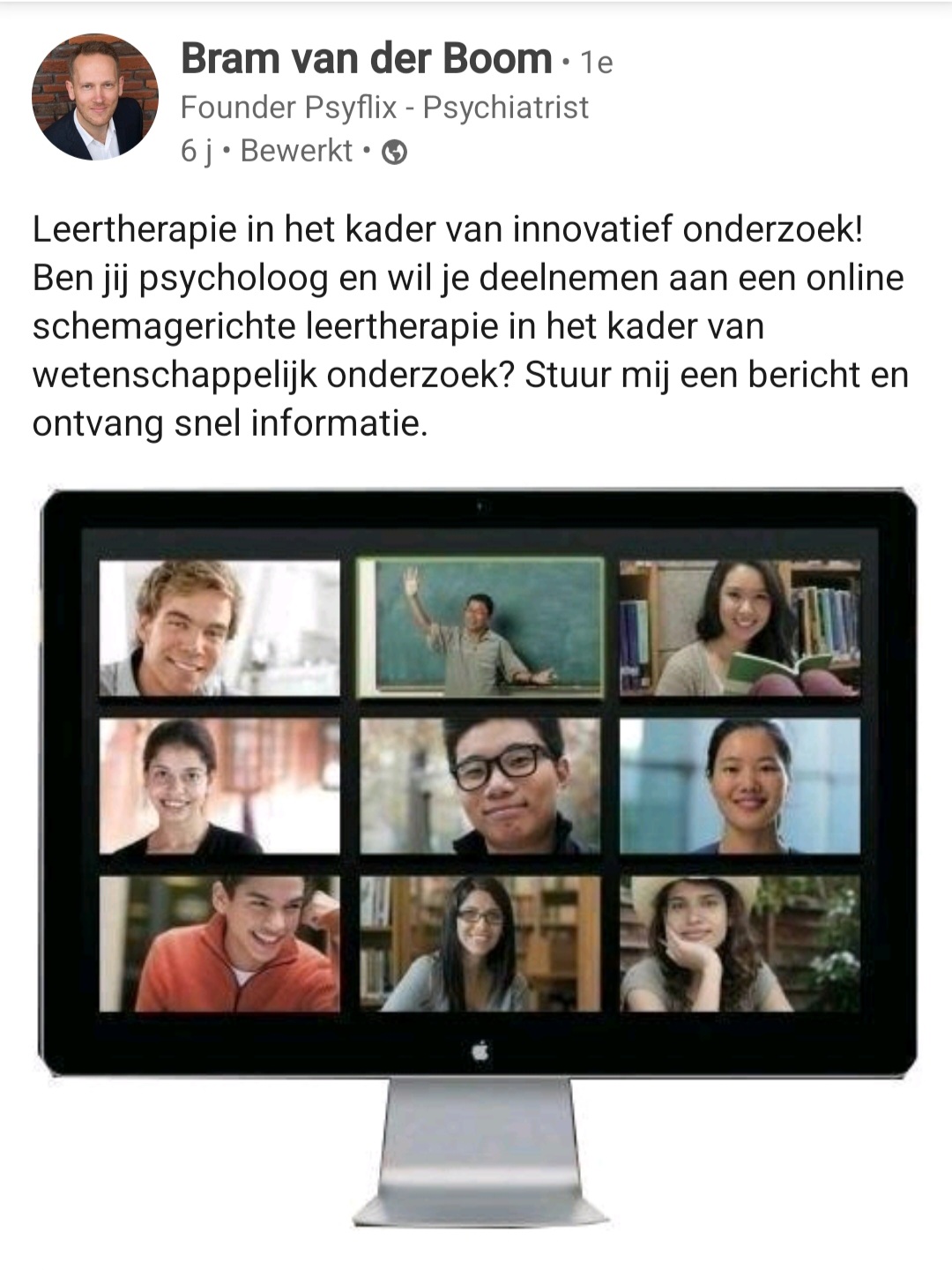


Training therapy as part of innovative research! Are you a psychologist and would you like to participate in an online schema-focused training therapy as part of scientific research? Send me a message and receive more information soon.
