# Supplementary material for: Feasibility of online group schema therapy: A preliminary study with therapists in training for future application in borderline personality disorder
Source: Internet Interv. 2025 Dec 5;43:100897. doi: 10.1016/j.invent.2025.100897 (PMC12768866; doi:10.1016/j.invent.2025.100897)
Supplement: Annex 2 — VC-GST Therapist Protocol. [file mmc2.docx]

**Protocol Online Group schema Therapy for Therapists**

Bram van der Boom and Pepijn Steures

- Introduction
- VC-GST
- Treatment Protocol (per session)

**Introduction**

This treatment protocol provides a structured, session-by-session guide for delivering online Video-Conferencing Group Schema Therapy (VC-GST) for individuals with personality disorders. It is designed to support clinicians in implementing an evidence-based, online adaptation of schema group therapy, with a balanced integration of both cognitive-behavioural and experiential techniques.

The development of this protocol is grounded in two core resources:

- *Kortdurende schemagroepstherapie, cognitieve gedragstherapeutische technieken, werkboek en handleiding - Vreeswijk & Broersen (2017).*
- *Werkboek kortdurende schematherapie: experientiele technieken - Vreeswijk & Broersen (2017)*

The content herein is adapted from these foundational texts. Certain components have been restructured and rephrased to enhance clarity and suit the online delivery format, while other sections have been directly incorporated. The combined model emphasizes therapeutic flexibility, clinical precision, and adaptation to digital therapeutic settings.

**Video-Conferencing Group Schema Therapy (VC-GST)**

The following sections provide theoretical and practical background information relevant to the delivery of online group Schema Therapy. A slightly modified version of this content is also provided to participants within their group workbook. While therapists may be familiar with conventional group therapy approaches, the transition to an online modality introduces specific dynamics that necessitate thoughtful adaptation.

Special attention is given to the psychodynamic foundations of schema therapy, particularly as they pertain to interpersonal processes in a group setting. Understanding these processes is essential for fostering therapeutic change within the group context.

**1. Traditional Group Psychotherapy (Face-to-Face)**

**1.1 General background** Group psychotherapy is a form of psychological treatment conducted in a group format, typically involving six to ten participants who meet weekly for sessions lasting approximately 90 minutes.

This therapeutic approach is generally appropriate for individuals who could also benefit from individual therapy. However, group therapy offers unique interpersonal learning opportunities through real-time interactions with others. Each group member brings their personal history, personality traits, and relational patterns into the group setting. These factors influence how they perceive and respond to others, as well as the roles they assume within the group dynamic.

Throughout the therapy process, participants gain insight into how they impact others and how others affect them. This self-awareness fosters personal growth and emotional resilience. The group provides a microcosm of the external world, where participants can experiment with new behaviours, receive feedback, and work through personal challenges in a supported environment.

**1.2 The role of the group psychotherapist** The group psychotherapist plays a facilitative, supervisory, and interpretive role. While the therapist may occasionally provide direction, pose reflective questions, or offer clarification, much of the therapeutic work emerges from the interactions among group members themselves.

The therapist’s primary responsibilities include:

- Establishing and maintaining group norms and boundaries
- Cultivating a safe, non-judgmental space where members feel emotionally secure
- Encouraging open communication and mutual respect
- Monitoring group dynamics and guiding therapeutic processes

In the initial phases, the therapist is more actively involved, modelling appropriate communication, setting the emotional tone, and helping to build cohesion. As the group develops, the therapist’s role becomes more supportive and process-oriented, allowing members to take increasing ownership of their therapeutic journey. The therapist remains vigilant in protecting the integrity of the group process and addressing potential disruptions.

**2. Video-Conferencing Group Schema Therapy (VC-GST)**

**2.1 Online group therapy: key considerations**Video-Conferencing Group Schema Therapy (VC-GST) mirrors many of the core principles of face-to-face group therapy but is conducted in a digital environment. This modality preserves the essential therapeutic functions of group work, interpersonal exploration, emotional expression, feedback, and corrective experiences, while offering increased accessibility and flexibility for clients.

To participate effectively, clients must have access to:

- A reliable computer (ideally no older than five years)
- A stable high-speed internet connection
- A functioning webcam and microphone
- A quiet, private setting where they will not be interrupted during sessions

These technical and environmental requirements are essential to ensure confidentiality, full participation, and a high-quality therapeutic experience.

**2.2 Interpersonal learning in groups**In both traditional and online settings, group therapy functions as a social microcosm, a condensed, emotionally rich environment where interpersonal behaviour can be observed, reflected upon, and modified in real time.

Interpersonal learning is one of the most powerful mechanisms of change in group therapy. Clients are encouraged to explore how they relate to others, how others perceive them, and how their behaviour affects the group. This exploration often brings unconscious patterns into awareness, allowing clients to practice more adaptive ways of relating.

Working directly on relationships within the group can provoke anxiety or resistance, but it is also where deep and lasting change occurs. By confronting interpersonal difficulties in a safe, therapeutic setting, clients gain insight and develop new relational capacities that can be generalized to life outside of the group.

Active participation and emotional honesty are key. Clients are encouraged to express their feelings, particularly those directed toward other group members or the therapists,in the moment they arise. This process fosters authenticity, emotional intimacy, and personal growth.

**2.3 Self-disclosure as a therapeutic tool**Self-disclosure is a central component of effective group therapy. It refers to the intentional sharing of personal experiences, thoughts, feelings, and memories with the group. These may include past events, current struggles, fantasies, dreams, hopes, fears, and interpersonal reactions.

Authentic self-disclosure facilitates deeper connection, both with oneself and with others. It allows group members to be seen and accepted as they truly are, laying the foundation for greater self-acceptance and emotional healing.

However, self-disclosure inherently involves risk. The more personal or emotionally charged the content, the greater the vulnerability. Clients often struggle with the fear of being judged, rejected, or misunderstood. The willingness to disclose is influenced by the perceived safety of the group environment, which the therapist must actively foster.

A non-judgmental atmosphere where active listening and mutual respect are the norm is crucial. The therapist is responsible for modelling and maintaining a group culture in which disclosure is met with empathy, curiosity, and support rather than criticism or dismissal.

**2.4 Giving and receiving feedback**Feedback is another cornerstone of group therapy. It provides clients with direct information about how they are perceived by others, offering insight into interpersonal blind spots, behaviours or attitudes they may not be consciously aware of.

Effective feedback is:

- Clear and specific
- Honest yet respectful
- Constructive and focused on growth
- Delivered with sensitivity to timing and emotional tone

Receiving feedback can be challenging, even when it is invited. It often evokes discomfort or defensiveness, especially when it touches on unresolved issues or areas of vulnerability. The therapist should prepare the group for this and help members navigate these moments with emotional maturity.

Providing feedback is also a vulnerable act. It requires courage and openness, as it may reveal the giver’s own sensitivities and limitations. Mutual accountability is key: both the giver and receiver bear responsibility for engaging in the feedback process constructively.

Feedback is most impactful when it balances confrontation with affirmation: highlighting areas for change while honouring the inherent worth of the individual. If delivered too harshly or provocatively, feedback can trigger fear or shutdown responses, undermining its therapeutic value.


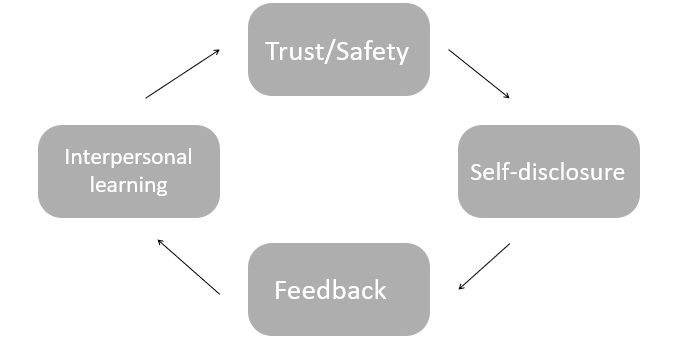


**2.5 The Johari Window**

The function of feedback and self-disclosure can be illustrated using the **Johari Window**, a model developed by psychologists Joseph Luft and Harrington Ingham (see Table 1). The Johari Window provides a graphic representation of interpersonal awareness and serves as a tool to better understand one’s relationship with oneself and others.

**Table 1.**

*The Johari Window*

|  | **Known to Others** | **Unknown to Others** |
| --- | --- | --- |
| **Known to Self** | Open Area | Façade |
| **Unknown to Self** | Blind Spot | Unknown (Unconscious) |

This framework highlights how group therapy can increase self-awareness by reducing both the blind spot (what others see but the self does not) and the façade (what the self hides from others), ultimately expanding the open area through feedback and self-disclosure.

**3. General agreements**

At the outset of Video-Conferencing Group Schema Therapy (VC-GST), a number of general agreements apply to all participants and therapists. These agreements are designed to support the therapeutic process and foster a safe and constructive group environment. While participation in group therapy is entirely voluntary, it is not without commitment.

The agreements we establish together serve as a foundation for emotional safety and mutual trust. This sense of security is essential to enable open and meaningful dialogue, where truth about the self and others can emerge and be explored.

**3.1 Confidentiality**

Confidentiality is of utmost importance in group therapy. Therapists are bound by professional ethical standards and legal confidentiality obligations, and group therapy is no exception. All information shared within the group will be treated with strict confidentiality. Therapists sign a confidentiality agreement to formalize this commitment.

Group members are equally expected to respect the privacy of others. This is essential for maintaining a safe space where sensitive and personal topics can be discussed openly. Therefore, participants are asked not to share any personally identifiable or sensitive information outside the group context, including through social media. This includes names, professions, and the personal stories or themes shared by other group members.

**3.2 Group conduct and interpersonal norms**

We aim to foster an atmosphere of openness, curiosity, and respect for differences throughout the group therapy process. Because secrecy fosters isolation, participants are asked not to form private subgroups or share confidential information among a few, excluding others. If interactions occur outside the group that could affect group dynamics, participants are encouraged to bring these experiences back into the group for open discussion.

The unique structure of group therapy often gives rise to strong emotional experiences, some of which may feel unfamiliar or intense. To process these emotions effectively, we encourage participants to explore and verbalize their reactions within the group, rather than acting impulsively on them. This is referred to as:
 "Feel it, but don’t act it out."

Alongside the relational aspect of group therapy, it is important to remember that the group also shares a collective purpose and therapeutic goal. The group is not intended to initiate new (particularly romantic) relationships, but rather to serve as a space for examining and transforming current relational patterns.

**3.3 Attendance and participation**

Group therapy functions as a coherent, cumulative process, each session builds upon the last. For the benefit of both the individual and the group, full attendance is essential. Naturally, there may be moments when attending feels difficult or confronting; these moments are often rich in therapeutic potential. We encourage participants to bring any hesitations, resistance, or frustrations into the group dialogue.

To avoid concern among group members, we ask participants to inform the therapists ahead of time if they will be absent from a session.

**4. VC-GST House rules**

The following house rules apply to all participants (and therapists) involved in video-conferencing psychotherapy. These are designed to create an environment that supports focused, respectful, and safe therapeutic work.

**4.1 Mobile devices**

To maintain a calm and distraction-free environment, please refrain from using your mobile phone during sessions. Phones should be placed on silent mode and remain visible, but unused.

**4.2 Food and drink**

We kindly ask that you refrain from eating or drinking during sessions to support presence and engagement.

**4.3 Alcohol and drug use**

The use of alcohol or drugs, either prior to or during the group session, is strictly prohibited.

**4.4 Recording**

Recording of any kind (audio or video) is not allowed. The video conferencing platform used is encrypted and secure; third parties cannot access the session.

**4.5 Quiet setting and neutral background**

Please ensure you are in a private, closed space during sessions where interruptions are unlikely (placing a sign on the door can help). The background should be neutral, without bright lights or visual distractions.

**4.6 Technical requirements**

A stable internet connection, high-quality sound, and a functioning webcam are essential for effective participation. For optimal performance, we recommend using a wired Ethernet connection rather than Wi-Fi whenever possible.

**4.7 Single-tasking**

Multitasking during sessions (e.g., browsing other websites or using other programs) is not permitted. This helps reduce distractions and preserves the quality of the connection.

**4.8 Camera position and framing**

Position yourself at a distance of approximately 50 cm to 1 meter from the camera. Your face and upper torso should be clearly visible. When speaking, try to look directly into the camera.

**4.9 Online communication guidelines**

Communication in an online group follows the same principles as in-person communication, with a few exceptions. We use specific hand signals to manage technical issues or signal the need for support:

- **Hand Signal 1: Raised hand** → “I would like to respond” (useful if you’re having trouble entering the conversation)
- **Hand Signal 2: Finger pointing to ear** → “I can’t hear properly”
- **Hand Signal 3: Two fingers to the eyes** → “I can’t see properly / screen is frozen or glitching”
- **Hand Signal 4: T-shape with hands (Time-out)** → “I’m being interrupted (e.g., someone entered the room)”

**Schema-focused Video-Conferencing Group Schema Therapy (VC-GST)**

This document outlines the structure, content, and therapeutic attitude recommended for schema-focused VC-GST. It is based on the manualized approach described in *Kortdurende schemagroepstherapie* (Vreeswijk & Broersen, 2017) and tailored for digital group settings. It includes guidelines for preparing patients, structuring sessions, and managing the group process across cognitive and experiential phases.

**Preparation and intake**

Before starting VC-GST, therapists assess whether patients are suitable candidates for schema-focused group therapy. This involves screening for relevant indications and counter-indications, sending out informed consent documentation, and administering the Young Schema Questionnaire (YSQ) and the Schema Mode Inventory (SMI).

Once all documentation and assessments have been returned, patients receive an individual intake session via Zoom. This session serves to introduce the therapeutic model, clarify the group format, and explore the patient’s motivation and goals. A follow-up session is held to finalise treatment planning. During this session, the treatment plan is reviewed, and patients sign the group rules and confidentiality agreements.

**Therapy structure and phases**

The VC-GST program consists of 20 sessions, divided into four distinct phases. Sessions 1 to 3 form the introductory phase, in which the group is formed and the cognitive foundations of schema therapy are introduced. Sessions 4 to 9 constitute the first working phase, which is primarily cognitive in nature. Sessions 10 to 16 make up the second working phase, in which experiential techniques are added. Sessions 17 and 18 are focused on closure and integration, while sessions 19 and 20 serve as booster sessions held at a later date.

**Session 1: Introduction**

The first session is dedicated to orientation and group formation. Therapists begin with a brief explanation of the rationale and goals of group therapy, emphasizing the importance of psychological safety and confidentiality. The practical structure of the group is explained, including the use of subgroups and how discussions within subgroups are brought back into the larger group context.

Following a round of introductions, patients are placed into pairs or triads to discuss the outcomes of their schema questionnaires and reflect on their personal goals for therapy. Each participant then introduces their conversation partner to the larger group.

The session continues with the introduction of a weekly mode-tracking document. Patients will use this document to monitor the activity and development of their dominant modes, particularly changes in the Healthy Adult and Happy Child modes.

A brief explanation of the schema and mode model is provided, supported by visual material. Patients are encouraged to begin identifying their own schemas and modes, even though these patterns often feel natural and are therefore difficult to recognise. The therapists explain that recognition will increase over time and that these patterns can eventually be challenged and changed.

Therapists normalize the emergence of strong emotions during therapy, clarifying that this is a sign of progress rather than danger. Emotional expression, both within and outside of sessions, is encouraged. Patients who report emotional numbing are also reassured that this too indicates change.

Homework for the following session includes reviewing psychoeducational material and reflecting further on personal schemas and therapy goals.

Therapists are advised to take an active but non-confrontational stance in early sessions. Any necessary confrontations should be delivered calmly and framed as part of the therapeutic process.

**Session 2: Schema exploration I (cognitive)**

The second session begins with a group discussion of the previous week, with particular attention to situations that activated schema or mode patterns. Therapists actively facilitate the conversation, helping participants reflect on emotional and behavioural responses.

The concept of schema avoidance, overcompensation, and confirmation is introduced using clinical examples. If possible, therapists should relate the material to real experiences shared by group members, reinforcing the understanding that these strategies perpetuate maladaptive schemas.

An experiential exercise follows in which participants are asked to draw representations of their dominant schemas and modes. They are given ten minutes for each. In pairs, they discuss their drawings and reflect on the feelings that emerged. The final part of the session is used to share these reflections in the full group, encouraging emotional openness and connection to underlying schema themes.

Therapists should remain attentive to the development of group dynamics and provide structure and containment as needed. Feedback should be focused on promoting understanding and empathy rather than early interpretation or challenge.

**Session 3: Schema exploration II (cognitive)**

This session follows a similar structure to session 2. The first part consists of a group discussion focused on schema activation in daily life. Therapists continue to facilitate active participation and encourage mutual reflection among group members.

Participants meet in subgroups to review their schema-monitoring homework, and key insights are shared in the plenary discussion that follows.

Toward the end of the session, participants complete a group climate questionnaire, which is then discussed in the full group. This promotes meta-reflection on the group process and helps to reinforce a shared sense of purpose.

If schema work remains abstract or emotionally distant, therapists may introduce a light-hearted warm-up exercise. For instance, the group can be invited to assign likely schemas to well-known public figures. This can introduce humour while still fostering insight. Therapist self-disclosure, used judiciously, may also help model openness and encourage participants to explore their own vulnerabilities.

**Session 4: Schema model I (cognitive)**

This session begins with a group discussion focused on recent experiences and schema activation. As in earlier sessions, therapists facilitate a structured reflection on emotionally significant situations, drawing attention to underlying schema themes. The emphasis is on connecting these situations to the Vulnerable Child mode. Participants are invited to reflect on what they would have needed in those moments, based on the five core emotional needs central to schema theory.

The core of this session involves the introduction of a case conceptualisation model. The therapist provides a brief instructional explanation on how to complete the model, ideally using personal examples. If self-disclosure is not appropriate, the sample case provided as a handout may be used instead.

Participants are then given time to begin filling out their own models, starting with the light blue sections (10 minutes), followed by identification of relevant schemas (1 minute), and then the light yellow sections (10 minutes). Participants are invited to complete the model by adding directional arrows between elements to illustrate the dynamic interaction of thoughts, feelings, and behaviours.

The group then reconvenes for an open discussion on the exercise. Participants are encouraged to share their experiences completing the model and reflect on any insights or emotional responses that emerged. Therapists introduce the idea that this conceptualisation will be revisited in the following session.

Therapists are advised to reflect with the group on the prevailing interpersonal style observed in the room (e.g., schema-compensating, avoiding, or confirming behaviours). The therapeutic response to these tendencies is named explicitly. For example, in the presence of overcompensation or dominating behaviour, the therapist may slow down the process and redirect focus to emotional awareness.

This can be introduced in a psychodynamic manner by posing an open question to the group: “What do you think is the dominant coping pattern of this group right now?” This facilitates meta-cognition and promotes group insight.

**Session 5: Schema model II (cognitive)**

The session begins with a 30-minute group discussion in which participants reflect on schema-related experiences from the previous week. Again, attention is drawn to the Vulnerable Child mode. Therapists ask participants to consider: What would you have needed in that moment? This reflection is explicitly tied back to the five basic emotional needs.

The main activity in this session is conducted in pairs. Participants share their schema models with one another in two rounds of 15 minutes. Therapists encourage curiosity, mutual questioning, and empathic listening. At the same time, it is important to affirm that participants are free to set boundaries, especially when it comes to discussing painful or sensitive life events. Privacy and emotional safety take precedence.

Following the pair work, the full group reconvenes to discuss their experiences with the schema model. Therapists invite reflections on how the exercise affected them emotionally and cognitively. Some participants may express a desire to confront individuals involved in the development of their schemas. Therapists can introduce the idea of writing an unsent letter to such a person as a way of expressing unresolved anger or pain. This is framed as an optional, therapeutic task to process emotional content rather than to take direct action.

Given that the group is now in the working phase, avoidance strategies may still be active. If avoidance is prominent, therapists are encouraged to use empathic confrontation to gently challenge the group and promote deeper emotional sharing. The goal is to help participants move beyond intellectualisation and toward affective engagement with schema material.

**Session 6: Evidence gathering (cognitive)**

As with previous sessions, this meeting begins with a group check-in focused on the past week and the schema-related challenges participants have faced. Therapists specifically ask about emotional reactions and coping strategies. Participants are encouraged to consider how the Healthy Adult mode can respond constructively to intense emotions. The importance of seeking support, comfort, and connection from others is also emphasised.

The central focus of this session is the introduction of the cognitive technique “evidence gathering.” Therapists explain the rationale and method: participants are asked to explore situations from their lives that either confirmed or contradicted one of their core schemas. The aim is to develop a more nuanced understanding of the origins and maintenance of maladaptive beliefs.

Participants are placed into subgroups to try out the exercise and begin identifying confirming and disconfirming evidence for one of their schemas. The therapist highlights that, although early experiences may have shaped core beliefs, later life experiences often provide alternative narratives that challenge the original schema.

Throughout the exercise, therapists interweave emotional interventions, asking questions such as “How does that feel for you?” and “What emotions come up when you hear this part of the story?” These affective prompts help to deepen engagement with the material and facilitate integration between cognitive insight and emotional processing.

**Session 7: Pros and cons analysis (cognitive)**

The session begins with a 30-minute group discussion. Participants reflect on situations from the past week in which schema-related patterns were activated. Therapists support emotional awareness by asking: How did you process feelings such as anger or sadness? How did you give those emotions space?

A cognitive technique is introduced: the pros and cons analysis. Participants are invited to explore the advantages and disadvantages of maintaining their core schemas. In subgroups, the exercise is trialed using examples from participants’ personal experience.

Change can be unsettling. Letting go of familiar patterns, even dysfunctional ones, can evoke fear. Schemas often resist change by creating anxiety and discouraging new behaviour. The therapist normalises this resistance and affirms the participant’s progress, emphasising the importance of continuing the change process.

By listing the pros and cons of specific schemas, participants are encouraged to develop insight into the internal logic of their patterns. The goal is to diminish the power of the schema and increase motivation to challenge it.

Therapists may also suggest doing the same exercise for modes, offering a similar analysis of their impact.

**Tip:** During the initial group discussion, ask participants whether they have observed any changes in themselves or others. Encourage them to reflect on shifts in behaviour or interpersonal dynamics. If helpful feedback is shared, invite those individuals to write it down for reinforcement.

**Session 8: Pie chart (cognitive)**

This session opens with a 30-minute group discussion, including a reflection on homework and schema activation in recent events. Therapists aim to hand more responsibility to the group, promoting self-regulation and ownership of the therapeutic process. If therapeutic intervention is needed, therapists reflect empathically on how schema-driven behaviour may influence group dynamics.

Next, the therapist explains the use of a pie chart (10 minutes). The purpose of this exercise is to gain a more realistic view of personal responsibility and challenge cognitive distortions often reinforced by schemas. Participants are asked to recall a situation in which they felt fully to blame, deeply rejected, or overwhelmingly victimised. They then assign percentage-based responsibility to themselves and to others involved (people or systems), using a pie chart to visualise the result.

Common schemas may lead to distorted beliefs such as: “It’s all my fault,” or “No one else understands what I go through.” This technique aims to disrupt that narrative and invite a broader, more balanced perspective.

The exercise is completed in pairs, with 10 minutes per participant. The group then reconvenes to discuss the insights generated by the pie chart. Particular attention is paid to feelings of guilt and shame. A core therapeutic aim is to help participants de-shame these emotions and move toward self-compassion.

**Tip:** When deeper personal issues are shared, the therapist ensures psychological safety by reminding group members of constructive feedback principles (e.g., using “I feel…” instead of “You are…”), and by reinforcing empathy and curiosity.

**Session 9: Mode model (cognitive)**

The session begins with a 30-minute group discussion, during which participants are invited to reflect on their functioning within the group. Therapists facilitate discussion on triggered schemas and resulting emotional modes. Key questions include: What happens inside you when a schema is activated? Which mode do you shift into?

The therapist then introduces the mode model, including the core goals of schema therapy: reducing the dominance of protective modes, deactivating punitive modes, validating the Vulnerable Child, and setting limits for the Angry Child. Over time, these shifts are intended to strengthen the Healthy Adult and nurture the Happy Child.

Therapists explain the model and answer questions, checking whether group members can recognise these modes in themselves.

The final 30 minutes of the session are used to complete the interim assessments: YSQ (Young Schema Questionnaire), SMI (Schema Mode Inventory), and any additional relevant forms. These instruments will inform the upcoming individual evaluation sessions and support further treatment planning.

At the close of the session, each group member is scheduled for a brief (maximum 30-minute) individual evaluation session with one of the group therapists, to take place before session 11.

**Session 10: Imagery (experiential)**

This session begins with a shorter, 20-minute group discussion focused on schema-related experiences from the past week. Therapists briefly check in on homework and emotional developments.

A short explanation (5 minutes) is provided regarding the upcoming imagery exercise. This is kept deliberately brief, as lengthy explanations can heighten anxiety. Participants are reassured that the exercise will be experiential and that they are invited to approach it with openness rather than performance.

The imagery exercise is diagnostic in nature. Its aim is to surface early unmet needs and make the connection between childhood experience and current schema patterns. The therapist guides the group through the following process:

1. Sit upright, feet flat on the floor, back supported.
2. Close your eyes and focus on your breath, notice the in- and outflow through your nose.
3. Allow recent memories to surface, moments of emotional difficulty in the past month. Let one significant memory emerge.
4. Step into that memory: what do you see, feel, hear? Who is present? What is happening?
5. Now ask: has a similar situation occurred earlier in life, particularly during childhood? Let yourself go back to that moment as a child.
6. Step into that scene: how old are you? What do you see, hear, and feel? Who is with you? What is their response?
7. When the memory becomes clear, slowly return to the present by focusing again on your breath. After a few deep breaths, count from 1 to 3 and gently open your eyes.

Instead of discussing the experience verbally, participants are asked to draw an image representing the memory and the emotional experience that surfaced (15 minutes).

In the final 30 minutes, the drawings and emotional experiences are discussed. Therapists help participants identify core unmet needs and reflect on how these needs could be addressed, both within the group and in their external relationships.

Participants are reminded to complete an evaluation form before the next session, which will be a group evaluation. They are also asked to bring three childhood/youth photos to the next session, each symbolising:

- the Vulnerable Child
- the Happy Child
- the Healthy Adult

**Tip:** It is recommended that therapists practice guiding the imagery exercise beforehand (e.g., record and review), to ensure appropriate pacing and a calming tone.

**Session 11: Evaluation**

This session focuses on evaluating the group’s progress. Prior to the meeting, each participant has had an individual conversation to review the outcomes of the interim assessment. As preparation, participants are invited to reflect on the key topics they wish to address during the evaluation.

The session begins directly with the evaluation, guided by the modes change list. The group discusses what members would like to explore or see in the remaining sessions, how they perceive the quality of relationships within the group, and which schemas they wish to continue practicing. This discussion is expected to take about 45 minutes.

Following this, the group works on a triptych exercise using photos participants have brought from their personal lives. This exercise is designed to increase awareness of their life histories and encourage sharing more about themselves with other group members. On a sheet of paper, participants place the photo representing their Vulnerable Child (VC) on the left, the Healthy Adult (HA) photo in the centre, and the Happy Child (HC) photo on the right. Under each photo, they write an “I-statement” in the present tense, describing what they feel in that particular mode. This part of the exercise takes about 15 minutes.

Next, each person presents their triptych to the group, briefly explaining their feelings and insights related to each mode, which takes approximately 15 minutes. The session ends with a 15-minute group discussion reflecting on the exercise.

**Session 12: Imagination with Rescripting- Experiential**

This session centres on the Vulnerable Child and consists of two exercises followed by a group discussion.

Begin by briefly revisiting the concept of the Vulnerable Child and the five basic emotional needs (approximately 5 minutes). Then proceed with the guided imagination and rescripting exercise as described below:

Invite participants to sit comfortably with their backs supported by the chair. Both feet should rest flat on the floor, and the spine held upright. Ask them to close their eyes and focus on their breathing. Encourage them to notice the sensation of air flowing in and out through their nose, simply observing each inhalation and exhalation.

Next, guide participants to turn their attention toward memories from the past month. Ask them to allow these memories to surface naturally, especially those involving difficult or painful emotions. Encourage them to bring each memory to mind one at a time.

Prompt them to notice if any memory stands out as particularly distressing, difficult, or emotionally charged with feelings such as fear, anger, sadness, or guilt. When such a memory arises, ask them to focus on it closely. Invite them to mentally re-enter that moment as if it were happening again, paying attention to what they see, feel, and hear, and whether they are alone or with others.

Once this recent memory is fully recalled, guide participants to explore whether there is a similar experience from earlier in their life, specifically from when they were a young child. Ask them to imagine that childhood situation in detail, how old they are, what surrounds them, who is present, what is said and done, how they respond, and what they feel emotionally.

At this point, introduce the Healthy Adult mode. Encourage participants to imagine themselves stepping into the scene as a compassionate and protective Healthy Adult. They offer comfort, set safe boundaries, and ask the Vulnerable Child what they need. Remind participants that the child is fully seen and accepted, allowed to feel their emotions without taking responsibility for others, and that the situation will be made safe and predictable. The Healthy Adult supports learning from mistakes, encourages self-expression and playfulness, and helps the child connect with their inner experience. Emphasize that the Vulnerable Child is not alone and does not have to struggle by themselves.

Ask participants to consider if the Vulnerable Child wants to say or ask anything to the people present in the memory, while the Healthy Adult remains supportive. They may take the time they need for this internal dialogue.

Finally, guide participants to gradually leave the scene and refocus on their breathing. Ask them to notice the rhythm of inhalation and exhalation once more. Prepare them for reopening their eyes by counting slowly to three. On the count of three, they may open their eyes, reorient to the room, and take a moment before responding or engaging.

Following the imagination exercise, conduct a related experiential exercise based on the chair technique, adapted for an online or seated setting (as all participants are already seated).

Ask one participant at a time to stand and then sit back down in their own chair, which is now designated as the "Vulnerable Child chair." While seated there, the participant embodies the Vulnerable Child from their imagination. They then select two fellow group members to represent the Healthy Adult mode. These two stand and take their seats, designated as the "Healthy Adult chairs."

The Vulnerable Child shares their imagined experience and feelings with the group. The Healthy Adults respond by expressing what they believe the Vulnerable Child needs in that moment. Allocate a maximum of three minutes per group member to ensure everyone has a chance to participate.

After the exercise, all three participants return to their original seats and roles.

Conclude the session with a group discussion reflecting on the experience. Encourage participants to share insights and feelings that arose during the exercise.

**Facilitator Tip:** If Healthy Adult group members become silent or unsure during their responses, do not hesitate to gently prompt or offer suggestions to help the process flow smoothly.

**Session 13: Imagination and Rescripting II- Experiential**

This session centres on painful memories that have recently been triggered. The imaginative exercise revisits the structure of the previous session but places more emphasis on the emotional pain being felt. The experience is guided by the emotion itself.

**Imaginative exercise**

Invite participants to settle comfortably into their seats. Ask them to sit upright, with their backs supported and both feet resting calmly on the ground. Gently instruct them to close their eyes and bring their attention to their breathing. Invite them to observe the air entering and leaving through the nose, breathing in… and out… in… and out…

Encourage them to let memories from the past month surface , moments when they experienced a painful or difficult emotion. Allow these memories to come up one at a time. Then ask:

- Is there a particularly painful or upsetting memory?
- Did a specific emotion overwhelm you?
- Is there a memory that triggered fear, anger, sadness, or guilt?

Direct their focus to the feeling that stands out most. Invite them to relive the situation as if it were happening again. What do you see? What do you feel? What do you hear? Are you alone, or is someone else there?

Return attention to the feeling itself. Encourage participants to hold it, name it softly, and repeat it to themselves three times. Let the feeling stay present for a moment.

Now, ask whether there has been a similar situation earlier in life that carried the same emotional charge. Did something like this happen when you were a small child? Can you recall a moment that felt just as painful or distressing?

Invite them to enter that memory. Visualize themselves as the child they were at the time. How old are you? What do you see? What sounds surround you? What emotions are present? Is someone else there, or are you alone? If someone is present, what are they doing? What are they saying? How are you responding? How does it feel?

Now introduce the presence of the Healthy Adult into the scene:

I am entering this moment as your Healthy Adult. I am here to protect you, to offer comfort, and to set safe, caring boundaries. What would you like me to do for you right now? What do you need from me?

I want you to know that I see you as a child. You are allowed to exist fully. You are allowed to be small. You are allowed to feel whatever you are feeling. You are not responsible for others around you. You are allowed to be a child.

I will make sure this situation becomes safe and predictable. I will help you understand that you don’t have to be perfect. It’s okay to make mistakes and to learn from them. You are allowed to express yourself, to be playful. I see you, and I will help you connect with what’s happening inside. You are not alone. You don’t have to face this alone.

Is there anything more you need from me as your Healthy Adult? It’s okay if you’re not sure. Just know that I will continue to stay in contact with you. I will be here for you.

Now ask participants to tune in again. What are you feeling now that the Healthy Adult is speaking to you in this way? What thoughts or emotions arise? As the child, is there anything you still want to say or ask to the people who were present in that memory? Is there anything you need to know?

Let them take their time to explore this.

Gradually, begin guiding them back to the present. Invite them to shift their focus to the rhythm of their breathing again , in and out. Let them take all the time they need.

Let them know you will count to three. At the count of three, they may open their eyes and gently reorient themselves to the present space. There is no need to respond right away; allow a moment for the transition. One… Two… Three…

**Follow-Up Writing Exercise**

After the guided imagination, participants are asked to write a compassionate message , something their Healthy Adult would like to say to the Vulnerable Child they encountered. This may reflect the words they heard during the exercise or something else that feels authentic and supportive.

**Chair technique (adapted for in-seat or online use)**

The adapted version of the “chair technique” will be repeated in this session. Each participant will again have a turn, with approximately three minutes per person.

One person assumes the role of the Vulnerable Child by symbolically sitting in the “child chair” (the same seat, now temporarily designated for this role). That person selects two fellow group members to serve as Healthy Adults, who then also move into their designated chairs.

The Vulnerable Child shares what they imagined and how it felt. The two Healthy Adults are invited to respond, offering verbal support or reassurance based on what they believe the Vulnerable Child needs.

Then, the person who had taken the Vulnerable Child role stands up and moves into one of the Healthy Adult chairs. They may now read aloud the warm and supportive message they wrote earlier, directing it to the child part of themselves they just represented. After the exchange, all three participants return to their original seats and roles.

To close, a group discussion will take place reflecting on the experience. Participants are encouraged to share any moments of resonance or recognition, and to explore how it felt to move between roles, from child to adult, from receiving care to offering it.

**Session 14: Historical roleplay- Experiential**

In this session, typically only two to three group members will have the opportunity to participate due to the depth and intensity of the exercise.

**Exercise introduction**

Participants are invited to close their eyes and recall a recent situation from the past week in which they experienced a painful emotion. Take your time to connect with the feeling.

Now consider whether this situation reminds you of a past experience , possibly something involving a parent, a school setting, or another close relationship. Reflect on whether this past event could be used as the basis for a role play.

It is important to note that this exercise is not intended to reenact a traumatic event. The goal is not to relive distress, but rather to develop new perspectives on personal schemas and coping patterns.

**Phase 1: Reenactment of the original situation**

You begin by playing yourself as a child in the situation you have chosen. Select a group member to play the role of your parent or significant other. The role play takes place in the present tense.

To help the other person embody the role, you provide them with five descriptive behavioral traits that reflect how this parent or other figure typically behaved. (This doesn’t need to be perfect , just enough to shape the role.)

At the conclusion of the role play, you are invited to share the core belief or emotional conclusion that surfaced during the interaction. This may reflect something central to your schema or current coping mode.

**Phase 2: Reversal of roles**

Now the roles are reversed. You take on the role of the parent or significant other, and the same group member plays you as the child. The aim is to empathize as deeply as possible with the person you are now portraying. Take a moment to consider:

- What stage of life were they in at the time?
- What were they dealing with emotionally or practically?
- Were they healthy, struggling, or preoccupied?

After the role play, reflect on the following questions:

- What stood out to you while playing the role of the parent or other figure?
- Did you notice anything about what they might have been feeling?
- Revisit the conclusion you reached in Phase 1 , has its meaning shifted?
- Do you believe it more or less now?
- As the parent figure, what do you notice when looking at the child in that situation?
- Could the child , given their age and dependency at the time , realistically have responded any differently?

This reflection helps shed light on how your past has influenced your present responses and allows space for compassion and re-evaluation.

**Phase 3: Trying a new response**

In the final phase, you return to the role of the child. This time, you will respond differently , trying out a new behaviour or way of expressing yourself that you previously discussed or imagined.

After the role play, take time to reflect as the original participant:

- Has anything changed in how you feel?
- Has your perspective shifted?
- Has your conclusion or belief evolved?

Then, both participants symbolically “step out” of their roles by briefly standing up, and return to their seats in their original group roles. This same process is repeated for one or two additional group members, depending on available time.

The last 20 minutes of the session are reserved for group sharing and discussion. Even those who did not participate directly are encouraged to reflect:

- What was it like to observe the role plays?
- Did anything feel familiar or resonate with your own experience?
- What insights did you gain , either through observing or imagining yourself in that situation?

**Session 15: The Demanding and Punitive Parent: Experiential**

This session begins with a 30-minute group conversation, as is customary. Participants are invited to reflect on experiences from the past week, including anything that may have surfaced since the previous session or in response to their homework assignments.

The session then turns toward the demanding and punitive parent modes. In small groups, each participant creates a visual representation, on a single A4 sheet, of their own internal demanding or punitive parent. This could be a drawing, a symbolic image, or words that capture the tone and content of this inner voice. After approximately 10 minutes of working individually, the drawings are presented to the larger group. Each participant then takes symbolic action to distance themselves from this mode, by tearing the paper, crumpling it, stepping on it, or another physical gesture of rejection. This act serves as an experiential way to begin emotionally disengaging from this harsh internal critic.

It is important to explore any resistance participants may feel toward this exercise. Some may fear that letting go of their punitive or demanding inner voice will result in losing discipline or motivation. This fear is understandable but misguided. The healthy adult mode is fully capable of maintaining structure, responsibility, and perseverance, without cruelty, perfectionism, or self-punishment. It sets expectations that are realistic and compassionate, not excessive or harsh.

The session closes with a 20-minute group discussion. Participants are encouraged to share how the exercise felt, whether anything shifted internally, and how they relate to the demanding or punitive voice now. Those who did not present are also invited to reflect on what they recognized or identified with in the process.

Facilitators are encouraged to highlight that confronting the demanding and punitive parent mode is a continuous inner task. It is something that must be actively challenged, corrected, or set aside on a daily basis. This is the role of the healthy adult: to offer discipline without punishment, structure without rigidity, and self-care without shame.

**Session 16: Angry Child- Experiential**

Anger is a difficult emotion for many group members. It is often suppressed due to various schemas and modes. As a result, people frequently bottle up their anger, which eventually erupts in the form of a rage outburst from the "angry child" mode. After such an outburst, many are startled and withdraw into a compliant, submissive state, only to explode again later. This cycle is a common pattern.

The session begins with a brief introduction on the function and value of anger. This is followed by a discussion of practical tips on how to express anger in a constructive and respectful manner. These tips are also included in the workbook. The aim of this session is to practice expressing anger in a way that reflects the healthy adult mode.

Key guidelines include:

- Begin early. Do not wait until you’re fully angry to speak up,start when you first notice irritation.
- Use “I” statements: for example, “I think…” or “I feel…”
- Clearly express what behavior from the other person affects you: “It affects me when you do this…”
- Don’t shy away from expressing vulnerable emotions: “I feel hurt or sad when you do this…”
- Be specific and focus on the current situation. Avoid vague language or delving into long-past events, which often leads to misunderstanding and frustration.
- Reflect beforehand on what you want to achieve by speaking up. Is your goal realistic?
- Remember that expressing anger should not be about retaliation. Expecting the other person to immediately change may not be realistic.
- Pay attention to non-verbal cues: your tone, volume, and body language matter.

Following this, the group engages in a structured role-play exercise.

Participants are first asked to close their eyes and reflect: Do I express irritation frequently or rarely, especially toward those closest to me? What might be my reason for this pattern? How was anger handled in my family growing up?

Next, as many group members as possible will participate in role-play. The first volunteer selects a fellow group member to play a close person (e.g., a partner, sibling, or parent). The initiating member briefly describes the situation and provides five behavioural traits of the person being portrayed.

The scene is acted out for a maximum of three minutes. The initiating member expresses their irritation as they typically would, using their usual mode. After the role-play, there is a short reflection: How did it feel? Which mode was active?

Roles are then reversed. The participant now plays the close person, and the other plays the initiating member. This second round also lasts up to three minutes.

While still in the reversed roles, a few questions are asked to the person playing the “close other”:

- Did you feel safe during the conversation?
- Were you able to understand the other person?
- Which mode do you think the other person was in?
- What would you have needed in order to feel seen and to better see the other person?

After the role-play concludes, another participant takes a turn. Once all group members have participated, a group discussion follows.

In this closing discussion, participants reflect on what they have learned from the exercise. Did they discover new ways of expressing anger? Did their experience of anger shift in any way? The aim is to foster greater awareness and healthier expression of this often-suppressed emotion.

**Session 17: Closure and the Healthy Adult**

This is the penultimate session, make sure to state this clearly to the group at the beginning.

Start by briefly discussing the *positive diary* and the potential impact it can have on participants’ emotional well-being and self-perception. Emphasize how recognizing positive experiences, however small, supports the development of the healthy adult mode.

A 45-minute group discussion follows. The theme of closure and saying goodbye should be addressed. If the group tends to avoid this topic, the therapist should bring it up explicitly. Invite participants to reflect on their feelings around the upcoming end of the group and whether it triggers any specific schemas or emotional responses.

Next, divide the group into two smaller subgroups to begin preparing for two assignments that will be completed before the final session:

- **Assignment 1:** Each participant is asked to purchase a card, one for themselves and one for each member of their subgroup. There is no need to write anything on the cards yet. The card should symbolically reflect the learning process you and the others have gone through in the group.
- **Assignment 2:** Each subgroup selects a short YouTube video (about five minutes) that they feel represents their experience in this therapy group. This could be symbolic, emotional, humorous, or reflective. It may be helpful to exchange email addresses so group members can discuss the video in the coming week. Please send the final video link to the group therapists one day before the last session.

**TIP:** Plan ahead and consider thoughtfully how you want to divide the subgroups, taking into account group dynamics and individual needs.

**Session 18: Final session**

In this final session, the group will reflect on the therapy process, complete two creative assignments, and look ahead to the follow-up sessions.

Begin with a brief group reflection on the therapy journey, what has been meaningful, what has changed, and what participants will take with them. Encourage open sharing.

**Assignment 1**In their subgroups, participants write cards: one to themselves and one to each of the other members in their subgroup. They have 10 minutes for this. Emphasize that these messages do not need to be perfect, focus on honesty and support.

After writing, the full group reconvenes. Each participant reads their cards aloud to the others in the large group. Once all cards have been read, they are collected and sent to the group therapists, who will forward them to the intended recipients after the session.

**Assignment 2**Each subgroup presents the YouTube video they selected to represent their group experience. The subgroups briefly explain why they chose that particular clip and what it symbolizes about their shared process.

Finally, the therapists provide a closing moment.

**TIP:** Therapists are encouraged to write a personal card for each group member. You may also consider showing a separate YouTube video, such as the short animated video about schema modes featuring the owl, as a final symbol of the therapeutic journey.

**Booster Session 1**

This session is shorter than usual, lasting 60 minutes, and is intended as a booster session rather than a full therapy session. The focus is on reflecting on the past month and encouraging participants to continue applying what they learned during the therapy process.

Begin with a group reflection on the past month:
What went well and what didn’t? When did old schemas or modes reappear? How could you reflect on these moments from the perspective of your Healthy Adult?

**Planning for the next two months**Participants break into pairs and each person takes 10 minutes (20 minutes total per pair) to create a personal plan for the upcoming two months. They may use tips from their workbook or develop their own strategies to support themselves from their Healthy Adult mode.

**Individual evaluation meetings**Each group member will also have a one-on-one evaluation session scheduled with a therapist. In this meeting, the results of the psychological questionnaires will be reviewed. The required questionnaires will be emailed to participants in advance.

**Booster Session 2**

This is the last time the group will meet. The session lasts 60 minutes.

Using the most recent test results and insights from each participant’s individual evaluation session, we will reflect together on the therapy process and consider what comes next.

As the final part of the session, each participant will create a personal maintenance plan. This plan serves as a monthly check-in tool, helping individuals reflect on their modes, schemas, and Healthy Adult. In pairs, participants will discuss their plans for 20 minutes and then briefly present them to the group.

**Tip:** Start this session by asking what has gone well in the past few months.
