# Supplementary material for: Feasibility of online group schema therapy: A preliminary study with therapists in training for future application in borderline personality disorder
Source: Internet Interv. 2025 Dec 5;43:100897. doi: 10.1016/j.invent.2025.100897 (PMC12768866; doi:10.1016/j.invent.2025.100897)
Supplement: Annex 3 — Patient workbook. [file mmc3.docx]

**Video-Conferencing Group Schema Therapy**

**Workbook for patients**

Bram van der Boom and Pepijn Steures

This workbook is based on two foundational texts that served as both model and inspiration for the development of this protocol:

- *Kortdurende schemagroepstherapie, cognitieve gedragstherapeutische technieken, werkboek en handleiding -* Vreeswijk & Broersen (2017)*.*
- *Werkboek kortdurende schematherapie: experiëntiële technieken -* Vreeswijk & Broersen  (2017)

**Introduction**

You are holding the *Video-Conferencing Schema Therapy Workbook*. This workbook is part of an online group therapy program designed to help you recognize and change the schemas and behavioral patterns that are holding you back in life.

Each session includes homework assignments, and the corresponding worksheets can be found at the back of the workbook.

We wish you strength, insight, and meaningful change as you progress through this therapy.

**Online Group Therapy**

**1. What is group psychotherapy?**

Group psychotherapy is a form of therapy conducted in a group setting. Eight participants meet weekly for 90 minutes. This type of therapy is suitable for anyone who might also benefit from individual therapy. What makes group therapy unique is that all members actively contribute to the therapeutic process.

In the group, interpersonal interactions reveal a great deal. Each participant brings their own personality, life history, and personal struggles. Based on this, they respond to others in specific ways and often adopt certain roles within the group. During therapy, you can begin to understand how others affect you and how you respond to them. These patterns often reflect how you relate to people outside the group as well.

Although individual members receive attention at different times, the focus remains on working through personal issues within the context of the group.

**1.2 What does a group psychotherapist do?**

Much of the progress in group therapy depends on the contributions of group members. While the psychotherapist may intervene at times by asking questions or offering insights, they generally encourage participants to speak freely.

The therapist is responsible for creating the right conditions for the therapy to succeed. They establish group norms and foster a safe, respectful, and open atmosphere, enabling members to express themselves honestly.

**1.3 Why Group Psychotherapy?**

One of the main benefits of group therapy is that participants realize they are not alone. While individual concerns may differ, most people struggle with common life themes.

Group therapy offers opportunities to recognize yourself in others, learn from one another, serve as role models, and support fellow group members. The interactions within the group often reflect broader life patterns, giving participants real-time feedback on how they relate to others.

**2. Online Group Psychotherapy**

**2.1 Online Group Therapy**

Online group therapy closely mirrors traditional in-person group therapy. However, the online format comes with a few additional guidelines.

To participate effectively, you’ll need a computer that is preferably no older than five years, a stable internet connection, a working camera, and a microphone. You must also be in a quiet, private space where you won’t be interrupted during sessions.

**2.2 Interpersonal learning**

Group therapy functions as a microcosm of real life, offering a unique setting for open and honest interpersonal exploration. This environment is intentionally structured to help you gain deeper insight into yourself.

Working directly on relationships with other group members is not always easy and can create tension, but it’s a crucial part of the process. Understanding and working through your relationships within the group helps you better understand your relationships outside of therapy.

You benefit most when you express your feelings openly and honestly in the moment, especially those involving fellow group members or the therapists.

This doesn’t mean that the group functions as a confessional. Each person is free to set their own pace and decide what they’re comfortable sharing. The group is a space to take emotional risks and experiment with new behaviours.

**2.3 Self-disclosure**

Self-acceptance is often preceded by being accepted by others. But to be accepted, you first need to let others see who you really are.

Successful participation in the group is largely determined by your ability to engage in self-disclosure. This might include sharing experiences from the past or present, fantasies, dreams, desires, expectations, or feelings toward other group members.

Self-disclosure involves taking risks, especially when sharing things you haven’t revealed before or that carry emotional weight. The longing for acceptance and understanding often coexists with fear of rejection, shame, or guilt.

The level of risk one is willing to take depends greatly on the response of the group. A safe atmosphere, one free from judgment, ridicule, or harshness, is essential. The group culture must support active listening and acceptance without quick judgment.

**2.4 Feedback**

Feedback gives you insight into how others perceive you. Many people have lacked positive feedback in their past, so receiving genuine appreciation in the group can be deeply healing.

That said, constructive and even confrontational feedback is also necessary. Through this, blind spots, behaviours or attitudes you’re unaware of, can be revealed. These insights help you grow, shift unhelpful patterns, and strengthen your interpersonal skills.

Group members share a mutual responsibility to offer feedback. The most effective feedback is both honest and respectful, confronting while also preserving the dignity of the receiver.

If feedback is too forceful or provocative, it may trigger fear and be difficult to process, diminishing its usefulness.

In the early stages, feedback often centres on first impressions and general feelings about the group dynamic. As members grow more familiar with each other, feedback becomes more individualized and behaviour-specific.

Since feedback from one person can be limited or biased, all members are encouraged to contribute. This creates a more well-rounded view of oneself and one’s interactions.

Good feedback is clear and actionable. Ideally, it should challenge and support at the same time. And just as important, the recipient must be open to receiving it.

Giving and receiving feedback requires a direct, open attitude. Even when welcomed, feedback can feel vulnerable. It may expose not just the recipient’s blind spots, but those of the feedback giver as well.

For this reason, everyone in the group carries shared responsibility for maintaining a safe and respectful feedback culture


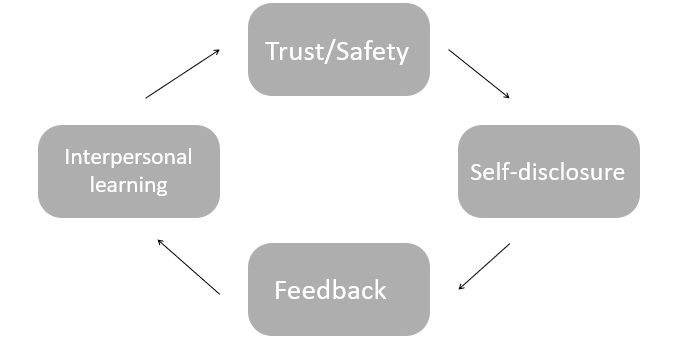


**2.5 The Johari Window**

The concepts of feedback and self-disclosure can be better understood using the Johari Window, a model developed by Joe Luft and Harry Ingham (see Table 1). The Johari Window is a visual representation of interpersonal awareness and can be a useful tool in understanding your relationship with yourself and with others.

|  | **Known to Others** | **Unknown to Others** |
| --- | --- | --- |
| **Known to Self** | Open Area | Façade |
| **Unknown to Self** | Blind Spot | Unknown (Unconscious) |

**Table 1. The Johari Window**

**3. General agreements**

To make the most of the time and energy we invest in each other, a number of general agreements apply to both therapists and participants. These agreements are designed to support and enhance the group process, and everyone is asked to commit to them.

While participation in group therapy is completely voluntary, it is not without obligation.

The agreements we make provide the structure and safety necessary to allow open and honest sharing, something essential for discovering truths about ourselves and others.

**3.1 Confidentiality**

Confidentiality is of utmost importance. Therapists are legally bound to professional confidentiality, and this also applies to group therapy. All information shared during sessions will be treated as strictly confidential. Therapists sign a confidentiality agreement.

Participants are also expected to respect each other’s privacy. This is critical for maintaining a safe space where sensitive topics can be discussed freely. Therefore, participants are asked not to share identifiable or personal information about other group members outside the group setting, including via social media. This includes names, professions, and any personal stories shared in the group.

However, you are free to talk outside the group about your own experiences and insights, as long as you don’t disclose anything about other participants.

**3.2 Group conduct**

We aim to foster a culture of curiosity, respect, and openness to differences. Because secrets create distance, we ask that no private conversations take place between group members about things that should remain hidden from others. If such conversations occur outside the group and are relevant to the group dynamic, we ask that they be brought back into the group discussion.

The unique nature of group therapy can evoke strong emotions, sometimes unlike anything you've experienced before. To explore these feelings productively, it's important to discuss them rather than act on them impulsively, a principle we call: "Feel it, don’t act it out."

This helps us remember that while our personal experiences matter, we also share a common goal. The group is a space to explore your relationships, not to form new (romantic) ones.

**3.3 Attendance**

Group therapy is a continuous process, where each session builds on the previous ones. Your presence is important not only for your own growth but for the group as a whole.

There may be moments when participating feels difficult. If this happens, we encourage you to discuss your concerns in the group rather than withdrawing silently.

To prevent concern from fellow group members, please inform the therapists in advance if you are going to miss a session.

**4. House rules for online video-conferencing group schema therapy (VC-GST)**

**4.1 Mobile phones**

To maintain focus and a calm environment, please do not use your phone during the session. Phones should remain on silent mode and kept nearby but not in use.

**4.2 Food and drink**

We ask that you refrain from eating or drinking during the group sessions.

**4.3 Alcohol and drugs**

The use of alcohol or drugs before or during the sessions is strictly prohibited.

**4.4 Recordings**

No audio or video recordings are allowed during the sessions. The video platform is encrypted and secure, preventing any external access.

**4.5 Quiet environment with a neutral background**

It’s important that each participant joins the session from a quiet, private room where interruptions are unlikely (a “do not disturb” sign on the door is recommended). The background should be neutral and free of bright lights.

**4.6 Stable connection, camera, and audio**

A stable internet connection and high-quality camera and microphone are essential. If possible, connect via Ethernet cable instead of Wi-Fi to ensure better stability.

**4.7 One task at a time**

Please do not run other programs or websites during the session, as this can cause distractions and technical issues.

**4.8 Framing and eye contact**

Position yourself 50 cm to 1 meter from your camera so your face and upper torso are visible. When speaking, look directly into the camera.

**4.9 Online communication**

Communication in an online group is similar to in-person interaction, but in some situations, we use hand signals to facilitate clarity:

- **Hand signal 1: raise hand** “I’d like to respond but can’t find a moment to speak.”
- **Hand signal 2: point to ear** “I can’t hear properly.”
- **Hand signal 3: two fingers to eyes** “I can’t see well; the video is frozen or unclear.”
- **Hand signal 4: make a ‘T’ shape (Time-out)** “Someone has entered the room or I’m being interrupted.”

**Schema Therapy**

Schema Therapy is a form of psychotherapy developed by Jeffrey Young, specifically designed to treat personality disorders. It integrates elements from Cognitive Behavioural Therapy (CBT), which focuses on symptom reduction, and psychodynamic therapy, which emphasizes self-awareness and emotional processing.

**Core emotional needs**

In Schema Therapy, it is believed that every child has several core emotional needs. When these needs are not adequately met during childhood, maladaptive schemas develop. These schemas become deeply rooted patterns that cause difficulties later in life.

Schema Therapy identifies six core emotional needs:

1. Safety
2. Connection & Belonging
3. Autonomy & Competence
4. Self-expression
5. Spontaneity & Play
6. Realistic Limits

When these core emotional needs are not adequately met during childhood, a child develops schemas. These schemas evolve into patterns that can cause difficulties later in life. Below, we will briefly discuss each of the core emotional needs.

| **Safety** A child needs to know that they are safe. Home should be a calm and reliable place where the child also has space to retreat. The child should not be exposed to sudden danger or illness, and if this does happen, there must be comfort and support available. | 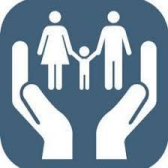 |
| --- | --- |
| **Connection** A child needs to feel connected to their parents and to other important people around them. The child must feel that they belong somewhere, within a family or a group, and that they are seen and known. In turn, the child should also be able to know and see others. | 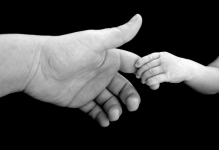 |
| **Autonomy** A child gradually needs more and more autonomy (doing things independently!). They must increasingly start doing things on their own in order to eventually reach adulthood. Too little or too much autonomy can be harmful and may lead to the development of schemas. Growing in autonomy fosters self-confidence and healthy relationships. | 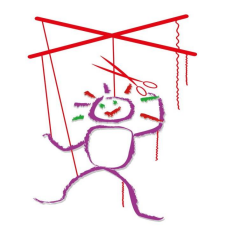 |
| **Self-expression** A child must be allowed to express themselves, both their opinions and their emotions. There should be space for anger, sadness, joy, and any other feelings the child experiences. A child needs to learn that emotions are not bad, but rather a tool for understanding how things are going and what is happening. In this way, the child learns to value their own emotions and integrate them into decision-making. | 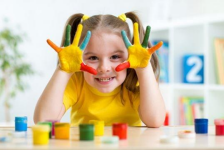 |
| **Spontaneity and Play** A child cannot always be focused on tasks, there must be moments when the pressure is off. There needs to be space for fun, spontaneous moments, for play, and for lighter experiences. Caregivers who fail to create space for this can inadvertently harm their child. | 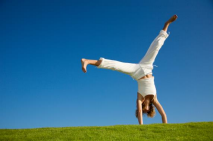 |
| **Realistic boundaries** Children seek boundaries and need to be given them. These boundaries must be realistic and appropriate to the child’s developmental stage. Through this, a child learns to understand themselves. They also learn not to act unkindly toward others, thereby developing their social skills.  Setting limits, for example, on how long a child works on homework, teaches discipline on the one hand, and on the other hand, helps the child learn when to stop before becoming exhausted. Learning to sense and recognize boundaries is an important developmental goal for children.  Children who receive no boundaries, or boundaries that are too strict, are more likely to develop maladaptive schemas. | 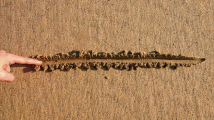 |

**Schemas**In schema therapy, we focus on three main components: schemas, modes, and coping strategies. We’ll go into detail about each of them.

Schemas are like filters, core beliefs through which you view relationships, yourself, and the world. A schema is essentially a deeply held conviction. Schemas develop when a child’s basic emotional needs are insufficiently (or excessively) met.

An example is the schema of *emotional deprivation* or *emotional neglect*.
With this schema, you typically believe that you will never receive the emotional support you need in life. When this becomes a core belief, it influences both your behaviour and emotional experiences. Below, you’ll find an overview of all schemas and their related beliefs.

**Coping strategies**Schemas are persistent and tend to become entrenched patterns in your life. They remain intact through different coping strategies. These are the three main types:

- **Surrender**You reinforce the schema by living as though it is true. For example, with the emotional deprivation schema, you may (unconsciously) choose partners or friends who are emotionally unavailable, thus confirming your belief.
- **Avoidance**You avoid situations where the schema might be triggered or felt. This way, the schema cannot be challenged or disproven. For example, with emotional deprivation, you might avoid entering relationships at all and choose to live in isolation. The schema remains unchallenged and intact.
- **Overcompensation**You become fed up with the schema and go in the opposite direction. You overcompensate for the painful belief, but in a way that paradoxically reinforces it. For example, in emotional deprivation, you might become overly demanding of your partner, constantly needing reassurance and emotional availability. If this continues, your partner may eventually leave, thus confirming your schema.
  You might recognize this confirmation by the internal voice saying: “See? This always happens to me.”

**Modes**A mode is a state of being, not a belief. It is a temporary emotional state that is triggered by certain situations and often comes with a fixed pattern of behaviour. Dysfunctional modes are frequently linked to schemas.

There are two key modes we aim to strengthen and develop:

- The Healthy Adult
- The Happy Child

In addition, there are other modes:

- Protector modes (defensive strategies)
- Parent modes
- Child modes

You’ll find an overview of these schemas and modes below.

| **Schemas** | **Description** |
| --- | --- |
| **Abandonment/Instability** | You expect to be abandoned by others. You perceive others as unreliable. |
| **Emotional Deprivation/Neglect** | You expect that you will ultimately not receive the emotional or practical support you need from others. |
| **Mistrust/Abuse** | You expect others to take advantage of you. You do not trust people and are on guard. |
| **Social isolation/Alienation** | You feel you don’t belong, like a stranger in a foreign land. As if you are different from others. |
| **Defectiveness/Shame** | You feel bad about yourself, see yourself as bad or not good enough. You fear that if others really see you as you are, they will reject you. |
| **Social undesirability** | You expect that you cannot keep up socially. You think you are unattractive and socially awkward. |
| **Failure** | You expect not to be successful in life. You anticipate failure in things you try, perhaps because you feel too stupid or clumsy. |
| **Dependence** | You expect not to be able to live life on your own, feeling very dependent on others to make decisions and handle things for you. |
| **Vulnerability to harm or illness** | You expect that you or those you care about can or will become ill or harmed at any moment. Or that some accident will happen. You feel powerless. |
| **Enmeshment/Undeveloped self** | You don’t know what your own feelings are or those of others. Often you are still ‘stuck’ to a parent, so your own identity hardly emerges. |
| **Self-sacrifice** | You believe you must sacrifice your own needs for others’. Possibly because others need it, or to gain their approval, or to avoid guilt or selfishness. This pattern often leads to anger. |
| **Subjugation** | You think that if you don’t submit to others’ wishes, you will be punished in some way. |
| **Unrelenting standards / Overly critical** | You think you always have to try harder or do more. You are critical of yourself and often of others as well. You are perfectionistic and critical, which makes it harder for you to enjoy things. |
| **Entitlement** | You believe you are better than others and deserve more than others. The rules that apply to others do not apply to you. Having power over others is often important to you in this schema. |
| **Insufficient self-Control / self-discipline** | You notice that you can endure and persist only a little. You have difficulty with discipline and self-control. You cannot tolerate setbacks and discomfort well. |

| **Mode** | **Description** |
| --- | --- |
| **Vulnerable Child** | You feel small and vulnerable, lonely, unseen, and unheard. You feel rejected or inferior. This feeling also influences your behavior and tone of voice. |
| **Angry Child** | You feel very angry and impatient because you don’t get what you need. You feel belittled or betrayed. You come across like a child having a tantrum , stomping and angry. |
| **Raging Child** | Similar to the angry child, but with little or no control over yourself. You become aggressive, hurtful, or damaging toward others. |
| **Impulsive Child** | You want something and want it immediately. You behave like a spoiled child and cannot tolerate waiting. You act without thinking like an adult would. |
| **Undisciplined Child** | You don’t want to do or finish tasks, you can’t tolerate the frustration of discipline, and you behave immaturely in this regard. |

| **Dysfunctional Coping Modes** | |
| --- | --- |
| **Mode** | **Description** |
| **Detached Protector** | You protect yourself from unpleasant feelings because you’re afraid you can’t tolerate them. You shut down and build a wall. Feeling “nothing” or emptiness is typical. You keep others at a distance and avoid the problem this way. |
| **Detached Self-Soother** | You prevent yourself from feeling unpleasant emotions by soothing yourself with familiar behaviors (e.g., shopping, eating, alcohol or drugs, excessive exercise, sex). This way you avoid the problem. |
| **Self-Aggrandizer** | You believe you are more entitled than others. You feel superior and behave accordingly. You brag, inflate yourself, and may belittle others. |
| **Bully and Attack** | You attack others before they can hurt or damage you. You intimidate others and want to maintain a dominant position. You enjoy attacking others. |
| **Demanding Parent** | In this mode, you believe you must always do your best and meet the highest standards and values. You can never do enough. You strive for perfection, often at the expense of enjoying life. You are never truly satisfied with what you do. This mode often corresponds to one of your parents. |
| **Punitive Parent** | You punish yourself. You believe you deserve it and you scold yourself or deny yourself things. You do not forgive yourself for your own mistakes. This mode often corresponds to one of your parents. |

| ***Healthy modes*** | |
| --- | --- |
| **Healthy Adult** | You look at yourself and others in a healthy, gentle, and pleasant way. You feel confident, but also recognize your own shortcomings without punishing yourself for them. You can see nuances and focus on the positive. You approach relationships in a constructive, honest, and open manner. |
| **Happy Child** | You want to play, have fun, and enjoy yourself. You feel seen, protected, and loved. You dare to try new things and come across as a free and happy child. |

**Treatment workbook per session**

**Session 1. Introduction**

In this session, you will get to know the other group members. Together, you will look at the results of your schema and mode questionnaires. Everyone will have a chance to briefly share why they started this group. For most people, this will be a bit nerve-wracking; keep in mind that it’s also nerve-wracking for others. You are not alone in this.

**Group rules:**We will also review the group rules in this session (see attachment).

**Registration:**Homework is an important part of schema therapy. One of the exercises we want you to do every week is to keep track of how things are going. We do this with two exercises.

- The first exercise is to write a short summary of each session: what went well and what did not (homework form 1).
- The second exercise is to keep track of your mode card. On this card, you list the three main modes you want to work on. Before each session, you fill out the card with how much trouble you had with each mode during the past week (score from 0 to 10; 10 means very much trouble, 0 means none) (homework form 2).

**Learning from the group:**In this group session, you will also hear from other participants which schemas and modes they struggle with. By listening to different stories, you will gain more understanding of schemas and modes, and how they work in your own life.

**Homework for next time:**

- Fill in homework form 1 (session summary)
- Fill in homework form 2 (mode card)
  Read the introduction text of this book regarding group therapy and schema therapy
- Read the text for session 2
- Prepare for session 2 by bringing paper and coloured pencils/markers

**Session 2. Schemas I**

Each therapy session begins with a group discussion of about 30 minutes. This is an informal conversation about what happened over the past week. It’s also an opportunity to refer back to the previous therapy session. The group therapists and members help you recognize your schemas and eventually change them. This all happens in an atmosphere of curiosity, safety, and understanding.
This is also a time to talk about any changes you have noticed in your tracked modes. In this session, we will further map out your schemas and modes.

We will do this with the following exercise:

1. Take a piece of paper (A4 size or larger) and some coloured pencils or markers. You will now have time to depict your three schemas. It does not matter how you depict or draw them. What matters is that you give a visual representation of what your schemas mean to you, what associations they evoke. Use as many colours as you want to express something.
   Take another piece of paper and do the same for your modes.
   You can also write text next to your drawings, jotting down as many associations as come to mind about your schemas and modes. It does not matter what you write; feel free.
2. If there is not enough time, you can finish your drawings during the week after the session. You can also create a new drawing or change the existing one. Schemas and modes will change over time.
3. Discuss in pairs what you have drawn, what it represents for you, and what it evokes. Feel free to ask questions about the other person’s drawing and exchange ideas.

It is common during this exercise to experience thoughts such as:

- "I can’t draw at all."
- "What a silly exercise."
- "Others can do it so much better and more beautifully than I can."
- "I’m not in kindergarten anymore; I won’t participate."

Realize that these thoughts might be influenced by schemas and modes that are active or being triggered. Schemas such as inferiority/shame, failure, ruthless standards/overly critical; modes like the demanding parent or detached protector might play a role.

As a result, you might become blocked. You might avoid participating in the exercise, try to make it perfect, or just scribble something without really engaging. It’s important to realize that schemas will always interfere with change. All change is scary, but try to participate anyway.

For the next 20 minutes, we will discuss in the group what representing the schemas and modes has meant for everyone. What did you think about? What did you feel? Did you notice anything in your body?

This will conclude the session.

**Homework for next time:**

- Read the text for session 3
- Homework form 1 (summary)
- Homework form 2 (mode card)
- Homework form 3 (mode registration diary)

**Session 3. Schemas II**

During this session, we will return to the mode registration diary. In the mode registration diary, you could write down a ‘dysfunctional’ mode, what you felt, thought, and did in that mode. The goal of this therapy is that these modes take less and less control in your life, and that you increasingly feel, think, and act from the healthy adult mode.

It is important that in this therapy, you are not only working on fighting the unhealthy modes but also on strengthening the healthy modes.

**Healthy Modes:**

**Healthy Adult**You look at yourself and others in a healthy, mild, and pleasant way. You feel confident but also see your own shortcomings without punishing yourself for them. You can bring in nuance and focus on the positive. You behave constructively, honestly, and openly in relationships.

**Happy Child**You want to play, have fun, and enjoy yourself. You feel seen, protected, and loved. You dare to try new things and come across as a free and happy child. The healthy adult mode can really help the vulnerable child (better than the protectors), truly giving what the child needs and genuinely protecting the child against the demanding/punishing parent. For this, the healthy adult knows what the vulnerable child needs.

**How to strengthen the healthy adult mode:**

**During the session:**Pay attention to how the therapists act and speak during the session. Do they come across as a healthy adult? What can you adopt from their behaviour, speech, and actions into your own life? Also, notice healthy adult behaviour from group members and give them a compliment.

**At home:**Write down one good thing you did each day and give yourself a compliment. A healthy adult rewards themselves (instead of punishing!).

Do one of the following activities:

- Read a good book, for example a novel by Dostoevsky or Yalom.
- Invite friends for dinner and cook yourself.
- Give someone a massage.
- Sit at a café terrace and observe the people passing by.
- Go to an auction.
- Go swimming.
- Visit a nice market.
- Go do photography, drawing, or painting.

**How to strengthen the happy child mode:**

**During the session:**Feel free to laugh and have a good time with your group, alongside the goals you want to achieve with therapy.

**At home:**Choose one of the following options and do it in the coming week:

- Watch a comedy movie or a thrilling series.
- Play a game (online or with friends at home).
- Throw a party.
- Sleep in well one day.
- Bake a cake or something else tasty.

**Homework:**

- Read the text for session 4
- Form 1 (Summary)
- Form 2 (Mode card)
- Form 3 (Mode registration diary)
- Fill in the group climate measurement form

**Session 4. Schema Model I**

**Groups and individuals:**

We each deal with our schemas in certain ways. We may avoid them, overcompensate for them, or even confirm them. These strategies allow the schemas to persist. Groups, as a whole, often also have a dominant way of coping with schemas.

Do you already have an idea of what the dominant coping style is in the group you’re part of? How could this be different?

**Schema Model:**

In this session, you will begin creating your own schema model , a comprehensive overview that connects the issues you're currently experiencing with your childhood. We’ll look together at what you were like as a child, how your parents were toward you, and possibly what it was like at school or with your siblings. Schemas arise when certain basic emotional needs of a child are not sufficiently met. These basic needs are:

1. **Safety**
2. **Connection** (together with safety, this creates attachment between parent and child)
3. **Autonomy**
4. **Self-expression and emotional expression of the child**
5. **Realistic boundaries according to the child's age**
6. **Spontaneity and play**

If these needs were not sufficiently present during childhood, schemas can develop. These schemas then lead to problems via different types of coping mechanisms, namely: surrender, avoidance, and overcompensation. These issues are likely the reason you are currently in therapy.

**Homework:**

- Read the text for Session 5
- **Form 1:** Summary
- **Form 2:** Mode card
- **Form 3:** Mode registration diary

**Session 5: Schema Model II**

In this session, we will revisit your Schema Model. This model is important because it helps you understand where your schemas and problems come from. When reflecting on this, strong emotions may surface, such as sadness, anger, regret, or guilt. It’s very important to talk about these feelings in the group. There's a high chance others in the group will recognize and relate to what you’re feeling.

Emotions like anger and sadness that come up are not meant to be pushed away.

- Anger can be expressed physically through exercise, movement, or even a combat sport.
- Sadness can be acknowledged and shared simply naming and feeling it can be healing.

**Example:**

Fedor realized after completing his Schema Model that he had a lot of anger toward how his father had treated him. He felt the anger physically as a constant tension in his body that wouldn’t go away.

A friend invited him to help chop wood for his fireplace. While chopping, Fedor noticed his anger starting to release. When he was done, he felt relieved, as though a weight had been lifted. For the first time, a part of his anger had found an outlet.

**Homework:**

- Read the text for Session 6
- **Form 1:** Summary
- **Form 2:** Mode card
- **Form 3:** Mode registration diary

**Session 6: Gathering information**

**Introduction**In this and the upcoming sessions, we will begin using several cognitive techniques to work on your schemas. Cognitive techniques help you think differently, specifically, to look at your schemas in a new way. They break the old patterns of thinking that have maintained your schemas. You will learn how to challenge and ultimately overcome them. It is important to take these techniques seriously and practice them consistently.

**The three cognitive techniques**

1. **Gathering information** – Looking at evidence for and against your schema.
2. **Pros and cons analysis** – Reflecting on what your schema has given you and the problems it has caused. What benefits and drawbacks could arise from changing the schema? In this technique, you list the positives and negatives.
3. **Pie chart** – This technique focuses mainly on schemas related to guilt, shame, responsibility, and perfectionism.

**Focus of this session: gathering information**Choose a schema you currently struggle with. Start by writing down all the evidence that supports this schema, proof that makes it seem valid or justified. Then, list evidence that contradicts the schema, things that weaken or disprove it. It’s important to examine whether the evidence applied only in the past or if it still applies now.

**What is a schema?**A schema is a belief about yourself, your life, or the world around you. These beliefs are not necessarily true. They may be completely false, or only partially or temporarily valid. To evaluate them objectively, you need to gather relevant information.

**Homework**

- Read the text for Session 7:
- Complete Form 1 (Summary)
- Complete Form 2 (Mode card)
- Complete Form 3 (Mode registration diary)
- Complete Form 4 (Gathering Information)

**Session 7: Pros and Cons analysis**

**Introduction**A cognitive technique used to change schemas is the pros and cons analysis. For each schema, you will explore the advantages and disadvantages of holding on to it. In the group, this will first be demonstrated using a schema that is common among participants.
Once you've practiced this with schemas, you can also apply it to the three dominant modes you struggle with.

**Why this technique helps:**To fight and change your schemas, you first need to understand them. You need to know why your schema is the way it is, and also what benefits it might bring. Schemas don't easily give up control, but once you clearly see the drawbacks, you’ll be more motivated to make a change.

**Example: Pros and Cons analysis,**  *Schema: Self-Sacrifice*

**Advantages of this schema**

1. Others like or love me and may look up to me.
2. Others need me and are less likely to leave me.
3. People don’t easily get angry with me because I sacrifice myself.
4. I feel good about myself, I’m not selfish, I care for others, maybe even better than others.

**Disadvantages of this schema**

1. I feel like I do more for others than they do for me.
2. My own needs and desires are not sufficiently met.
3. I sometimes feel angry or resentful toward others.
4. I feel responsible for others, even when I’m not.

Now imagine changing and/or healing the Schema/Mode.

**Advantages of change**

1. I take better care of myself.
2. More equal relationships.
3. Less anger and resentment.
4. Lower risk of burnout.

**Disadvantages of change**

1. My self-image might suffer.
2. Others won’t feel indebted to me anymore.
3. I’ll feel more afraid of people getting angry or abandoning me.
4. I’ll have less to do and will have to focus on myself more.

**Homework for next time**

- Read the text for Session 8
- Complete Form 1 (Summary)
- Complete Form 2 (Mode card)
- Complete Form 3 (Mode registration diary)
- Form 5 (Create a pros and cons analysis for your schemas and modes)

**Session 8: Pie Chart**

We are almost halfway through all the sessions and will soon take a moment to reflect. Now is a good time to begin looking at what you’ve already learned and what may have started to change. In this session, we will work with the final cognitive technique: the pie chart. This method helps you assess how much responsibility or guilt you actually bear in a given situation. Many schemas cause you to feel overly responsible or guilty, leading you to carry more blame than is fair or helpful.

**The purpose of guilt**

Freud once called guilt the most harmful of all human emotions. This is because some people completely collapse under the heavy burden guilt can impose. Guilt often causes intense and sometimes unnecessary suffering. Of course, everyone feels guilty now and then, but it shouldn’t be a daily part of life. Many people never learned how to deal with guilt properly,often, it was even made worse by their parents.

That’s why it’s important to work through guilt using the following three steps:

1. **Step 1**: Ask yourself: Do I truly feel guilty in this case? Did I really do something wrong?
   - If the answer is yes, go to step 2.
   - If the answer is no, go to step 3.
2. **Step 2**: Can I do something about it? (e.g., behave differently, apologize, etc.)
   - If yes, then do that. Then go to step 3.
   - If no, still go to step 3.
3. **Step 3**: Recognize that the guilt no longer serves a purpose. It’s no longer useful. You are allowed to let it go,or even actively release it. You can make room for other emotions.

**Using a pie chart against schemas**Pie charts are especially useful for schemas that make you feel responsible or guilty for certain events or situations. The pie chart visually helps distribute responsibility across different people or factors, instead of placing it all on yourself.

**Example pie chart** *Schema*: Unrelenting standards / overly critical
 *Thought*: “My parents are unhappy because I don’t visit them often enough.”
 *Schema credibility (0–100)*: 95%

**List of contributing factors (minimum 4)**

1. They are elderly and frail
2. My brother doesn’t visit often either
3. They have a poor pension
4. They’re losing many friends to cancer and other illnesses
5. They live in a house that’s too big to maintain
6. They’re disappointed in what they’ve accomplished in life
7. They watch the news daily and are affected by global negativity
8. My father lost his driver’s license and rarely goes out

Now, assign each of these factors a piece of the pie, including your own contribution (e.g., “I don’t visit often”). After this distribution, you’ll often find your own share is much smaller than you initially felt.

*New schema credibility (0–100)*: 25%

**Homework**

- Read the text for Session 9
- Complete Form 1 (Summary)
- Complete Form 2 (Mode card)
- Complete Form 3 (Mode registration diary)
- Complete Form 6 (Create a pie chart for one schema)

**Session 9: Modes**

In this session, the focus will shift more toward modes. A mode is a state of mind, an emotional state that you shift into when a schema is triggered. The more awareness you develop about which mode you're in at a given moment, the more opportunities you'll have to change it.

From Session 9 onward, we will place greater emphasis on experience and emotion. You will also receive a package at home containing a few items we’ll use during exercises.

The goal of therapy is to care for and, when necessary, set boundaries for the vulnerable child. To do this, we must first dismiss the demanding and punitive parent modes. After that, the protective modes are reduced or softened.

It’s important to realize that these protectors were once helpful, they got you through difficult times and helped you survive to this point. It can sometimes even be helpful to thank them. But you no longer need them, not in the group and not outside it. These are old coping strategies you learned as a child. While they once protected you, they now mostly serve to repeat patterns you no longer want.

When the vulnerable child receives the care it needs, it naturally begins to grow and gradually becomes a healthy adult. As an adult, you can learn to deal with your problems in an adult way, this is the *Healthy Adult Mode*.

**Homework**

- Read the text for Session 10
- Have paper and coloured pencils or markers ready again for next time:
- Complete Form 1 (Summary)
- Complete Form 2 (Mode card)
- Complete Form 3 (Mode registration diary)

**Session 10: Imagery**

**Emotional core needs**The emotional core needs mentioned earlier are essential needs that every child has. Just as your body needs healthy food to grow, your soul, your *psyche*, needs emotional nourishment.
A helpful metaphor for this is a child’s nutritional needs. When a child gets too little vitamin D, for example, they can develop a disease called rickets. This condition leads to soft bones and bowed legs.
Psychologically, the same principle applies: if we don’t get enough “psychological vitamins”, emotional core needs, our emotional development also becomes distorted.
Now is the time to become more aware of the emotional needs you have. This awareness will help you ensure that, in your relationships with yourself and with others, these unmet needs can finally be fulfilled. That is an essential step in healing your schemas.
Here again are the emotional core needs of a child:

- Safety
- Connection (along with safety, this forms secure attachment)
- Autonomy
- Self-expression and emotional expression
- Realistic limits appropriate to age
- Spontaneity and play

| **Safety** A child needs to know that they are safe. Home should be a calm and reliable place where the child also has space to retreat. The child should not be exposed to sudden danger or illness, and if this does happen, there must be comfort and support available. | 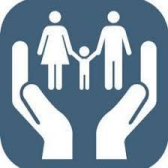 |
| --- | --- |
| **Connection** A child needs to feel connected to their parents and to other important people around them. The child must feel that they belong somewhere, within a family or a group, and that they are seen and known. In turn, the child should also be able to know and see others. | 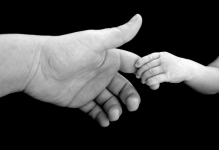 |
| **Autonomy** A child gradually needs more and more autonomy (doing things independently!). They must increasingly start doing things on their own in order to eventually reach adulthood. Too little or too much autonomy can be harmful and may lead to the development of schemas. Growing in autonomy fosters self-confidence and healthy relationships. | 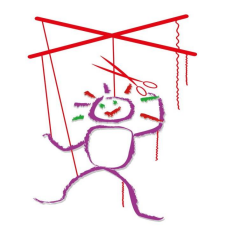 |
| **Self-expression** A child must be allowed to express themselves, both their opinions and their emotions. There should be space for anger, sadness, joy, and any other feelings the child experiences. A child needs to learn that emotions are not bad, but rather a tool for understanding how things are going and what is happening. In this way, the child learns to value their own emotions and integrate them into decision-making. | 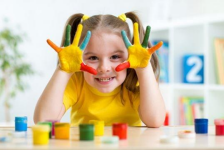 |
| **Spontaneity and Play** A child cannot always be focused on tasks, there must be moments when the pressure is off. There needs to be space for fun, spontaneous moments, for play, and for lighter experiences. Caregivers who fail to create space for this can inadvertently harm their child. | 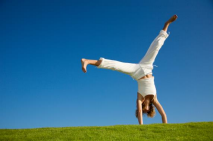 |
| **Realistic boundaries** Children seek boundaries and need to be given them. These boundaries must be realistic and appropriate to the child’s developmental stage. Through this, a child learns to understand themselves. They also learn not to act unkindly toward others, thereby developing their social skills.  Setting limits, for example on how long a child works on homework, teaches discipline on the one hand, and on the other hand, helps the child learn when to stop before becoming exhausted. Learning to sense and recognize boundaries is an important developmental goal for children.  Children who receive no boundaries, or boundaries that are too strict, are more likely to develop maladaptive schemas. | 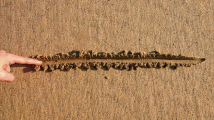 |

**Imagery exercise**In an imagery exercise (Genderen & Arntz, 2010), you mentally visualize a situation. You pause and reflect on what you feel and experience in contact with others or when you're alone.
Today, we’ll do an imagery exercise in the group to explore how you’re currently feeling, how this connects to your past, and how it relates to your schemas and emotional core needs. You’ll be asked to close your eyes while the group therapist gives simple verbal instructions.

Everyone will participate, and the focus won’t be on one individual. During the exercise, no one is expected to answer out loud. You are invited to focus on your own inner experience.

**Visual expression**After the imagery, you’ll be invited to draw something that reflects your experience. It doesn’t matter *how* you draw or *what* you draw, what matters is the image or feeling that arose during the exercise.

**Reflection**In the final part of the session, we’ll reflect on the imagery exercise. You may show your drawing. Are your emotional core needs starting to become clearer? Can the group offer support in meeting them?

**Homework**

- Read the text for Session 11
- Form 1 (Summary)
- Form 2 (Mode card)
- Form 3 (Mode registration diary)
- Bring childhood photos to the next session: one showing your vulnerable child, one of your happy child, and one of your healthy adult.
- Form 7 (Evaluation form)

**Session 11: Evaluation + Photos**

In this session, the group will reflect on how the therapy is going so far. What have you learned up to this point? In what ways has the group grown, and what areas could still use attention in the coming sessions? What are your personal goals for the second half of the therapy?

During childhood, we go through experiences that leave a deep emotional imprint, both positive and negative. By shedding light on different aspects of these experiences, we can form a clearer picture of our youth and of ourselves. Sharing this with the group also allows others to understand us better.

In this session, we’ll use personal photos to tell, and more importantly, show, each other something about our childhoods. For each photo, write a word or a sentence that captures the feeling that this particular photo evokes in you.

Share your experiences and your photos with the group. Notice whether you recognize something of yourself in what others show and share.

**Homework**

- Form 1 (Summary)
- Form 2 (Mode card)
- Form 3 (Mode registration diary)

**Session 12: Imagination and rescripting I**

In this session, you will do two exercises focused on the vulnerable child part of yourself.

**Basic needs**Every (vulnerable) child has a number of core emotional needs. Schema therapy identifies six:

- Safety
- Connection
- Autonomy
- Self-expression
- Spontaneity & play
- Realistic boundaries

In these exercises, you'll begin learning how to receive care, and possibly, how to give care to yourself.

The first exercise is another imagination exercise. The only new element is that the therapist may now introduce “rescripting,” where an alternative version of a past event is imagined. This helps you better recognize your needs and feelings.

The second exercise focuses on one group member at a time. The person in focus chooses two fellow group members to act as "healthy adults." You start by expressing what you experienced as the vulnerable child during the imagination exercise. Try to speak in the present tense and from an “I” perspective. Describe what you are feeling.

Afterward, the two healthy adults may respond to you with supportive words. You don’t need to reply, it’s mostly about letting the words sink in. The group therapists may also assist. Each group member will spend 3 to 5 minutes in this exercise.

The group will then debrief and reflect on the experience together.

**Homework**

- Form 1 (Summary)
- Form 2 (Mode card)
- Form 3 (Mode registration diary)

**Session 13: Imagination and rescripting II**

Today’s focus is on painful memories. These memories can be triggered in various situations, causing us to enter different modes. To gain better control over these painful memories and to soften their impact, we will do another imagination exercise. The emphasis will be less on the situation itself and more on the feelings involved.

After the imagination exercise, one group member will be the focus. You may choose two group members to act as two healthy adults. You begin by expressing what you experienced as your vulnerable child during the imagination exercise. Try to put it into words in the present tense and from an “I” perspective, describing what you feel. Once you have done this, the healthy adults will respond with supportive words. You don’t need to reply; the goal is to let their words reach you. The group therapists may assist as needed.

Next, you will be asked to stand and then sit back down in the same chair, but now as the healthy adult. Read a warm, supportive sentence aloud to your vulnerable child and let it sink in.

**Homework**

- Form 1 (Summary)
- Form 2 (Mode card)
- Form 3 (Mode registration diary)

**Session 14: Historical role play**

In this session, we will do several role plays. These role plays help you experience a different perspective on past situations. By doing a role play, a schema can often be changed, it can be weakened or nuanced and thus permanently altered.

A historical role play consists of three parts or phases:

- Phase 1: You act out a past situation exactly as it happened.
- Phase 2: The roles are reversed, you play your parent or caregiver, and another person plays you as a child.
- Phase 3: You consider if your child-self could have acted differently, and then act out that alternative behaviour. It’s important to note that this is not done because we think you should have acted differently as a child. Rather, the insights gained here can help you in your current relationships.

**Tip:** Speak in the present tense during the role play to make it feel more real and impactful.

**Homework**

- Form 1 (Summary)
- Form 2 (Mode card)
- Form 3 (Mode registration diary)

**Session 15: Demanding and punitive parent**

The demanding and punitive parent parts prevent you from properly attending to your sensitive feelings and taking good care of yourself. You may feel irritated daily and your emotions don’t get enough attention. In the punitive parent mode, you often get angry at yourself, while your vulnerable child is neglected.

Acting from these modes usually happens automatically. They can feel so natural and “right” that it’s hard to recognize them as separate modes.

We will now divide the group into smaller subgroups. Together, you will work on your own demanding and punitive parent on a sheet of paper. Use also the aspects others have mentioned. Add texts or statements that the demanding or punitive parent says to you.

Then, in the full group, the modes will be shared and discussed. We will think together about how to deal with these modes. Some might want to tear them up, others might crumple them into a ball. The important thing is to symbolically get rid of them. Many people fear that if they get rid of the demanding parent, they will become undisciplined. This is a misconception. The healthy adult has realistic expectations of themselves. They do require perseverance and discipline at times, but not excessively and certainly without the punitive side.

**Homework**

- Form 1 (Summary)
- Form 2 (Mode card)
- Form 3 (Mode registration diary)

**Session 16: The Angry Child**

We are nearing the end of therapy, with just two sessions left.

The role of anger in modes: Anger is a powerful emotion. It can destroy things, cause others to see you as less, or lead to abandonment. Because of this, anger is often suppressed by various schemas and modes. People may suppress their anger until it erupts as an outburst from the raging child mode. Shocked by this, they may then shift into a compliant, powerless state, only to later explode again. This is a common pattern.

How do you express anger in a healthy, respectful (healthy adult) way? That’s what we will practice in this session.

First, start early, not by talking about anger when you’re already angry, but by addressing irritation as soon as you notice it. Express irritation using “I” statements, for example, “I find it upsetting when you speak to me like that,” or “I don’t think this is a good idea and I don’t want to continue.” Say what in the other person’s behaviour affects you, e.g., “It hurts me when you do that.” Don’t be afraid to share vulnerable feelings: “I feel sad or vulnerable when you do that.”

Be as clear and specific as possible about what is happening now. Vague statements or bringing up very old issues often cause misunderstanding or irritation. For example, “What upset me was when you just did this…”

Think beforehand about what you want to achieve by expressing your irritation and whether that goal is realistic. It’s important that venting your anger is not your only goal. Some expect others to change just because they express their anger, which isn’t always realistic. Pay attention to your non-verbal behaviour: your tone, volume, and body posture.

In this session, we will also do role-plays around the theme of anger, guided by the therapists.

**Homework**

- Form 1 (Summary)
- Form 2 (Mode card)
- Form 3 (Mode registration diary)
- Practice expressing irritation during the coming week.
- Choose music for next week’s session to play quietly in the background (using headphones) that you as a healthy adult truly enjoy.

**Session 17: Closing and healthy adult**

This is the second-to-last therapy session. We prepare for ending the therapy. To keep growing and staying motivated, it’s important to encourage yourself. We do this by keeping a positive diary. In it, you write daily about the victories you’ve achieved over your schemas and modes. You can write multiple victories, but at least one per day. Do this in the coming week and continue for at least four weeks after therapy to build it into a habit.

The healthy adult is a mode that needs to be strengthened. Giving and receiving compliments is part of this. We will now divide the therapy group into two smaller groups. Everyone is assigned to buy a card for themselves and for the other members of their subgroup. You don’t have to write anything on it yet. Choose a card that symbolizes the learning process you and the others have gone through in this group.

Together with your subgroup, pick a YouTube video of about five minutes that represents your experience in this therapy group. It might be helpful to exchange email addresses so you can discuss it in the coming week. Send the video link to the group therapists one day before the last session.

**Homework:**

- Form 1 (Summary)
- Form 2 (Mode card)
- Form 3 (Mode registration diary)
- Form 8 (Write daily in your positive diary)
- Buy a card for yourself and the others in your (sub)group. Think about what you want to write on it.
- Find a YouTube video of max 5 minutes and send the link along with your subgroup to the group therapists.

**Session 18: Closing**

After this session, you still have two follow-up sessions. Today you reflect on what you have learned. We also look ahead to the coming weeks when you will no longer have weekly therapy groups. We will close this group with a ritual.

In the coming phase, it is important to hold on and keep practicing what you have learned. Schemas are persistent, but persistence pays off.

**Assignment 1:**In this session, write a card for the people in your subgroup within 10 minutes. Write a card to yourself and cards to the other members of your subgroup (don’t worry about perfection). Afterwards, everyone returns to the full group. The cards will be read aloud to each other in the large group. After this session, the cards will be sent to the group therapists, who will forward them to the respective group members.

**Assignment 2:**Both subgroups will show their chosen YouTube video clip. Each subgroup may explain their choice.

**Closing:**In the last 20 minutes, we focus on closing the therapy. The dates for the follow-up sessions will be mentioned again.

**Booster session 1**

This 60-minute booster session is designed as a brief check-in to help you reflect on your progress and strengthen the skills you’ve developed in therapy. While not a full session, it provides an opportunity to reconnect with your Healthy Adult mode and prepare for the months ahead.

We’ll begin with a group reflection on the past month. What went well? What challenges came up? Were there moments when old patterns or modes returned, and how did you respond, or how might you respond differently next time, using your Healthy Adult perspective?

Following this, you’ll work in pairs to create a personal plan for the next two months. Each person will have about ten minutes to consider strategies that will support their ongoing growth. You may draw from your workbook or use your own insights and experiences.

In addition, each group member will have an individual evaluation meeting with a therapist. During that session, you’ll review your progress and discuss the results of the psychological questionnaires. These will be emailed to you in advance for completion.

While brief, this session is an important part of maintaining your progress. Continued practice and reflection are key.

**Booster Session 2**

This is the final meeting of the group, and the session will last 60 minutes. Together, we’ll take time to reflect on the overall therapy process and look ahead to the future, using insights from your recent evaluation sessions and questionnaire results.

To begin, we’ll check in as a group by exploring what has gone well over the past few months. This helps set a positive tone and highlights the progress each of you has made.

The focus of this final session is creating a personal maintenance plan. This plan is designed to support you in the long term. It is a tool you can revisit monthly to reflect on your modes, schemas, and the perspective of your Healthy Adult. You’ll work in pairs for about 20 minutes to discuss and shape your plans, and then each person will briefly share their approach with the group.

Although this marks the end of group sessions, the process of growth continues. This session is about helping you to continue to move forward with confidence and clarity.

**Appendix 1:**

**Group Rules**

1. Confidentiality is very important for every participant so that they dare to share something personal in the group. Everything said in the group stays in the group. Do not discuss it with anyone outside. If you talk about group matters, never mention the names of group members.
2. Your presence is important. If you arrive late or in exceptional cases cannot attend a meeting, please let us know before the group starts. You can notify the therapists by phone; if they are unavailable, you may use email.
3. The use of alcohol or drugs before coming to the group is not allowed. If you have used them, you cannot participate in that session.
4. During group discussions, strong feelings may arise. It is important to express these feelings in a way that does not threaten others in the group. If you repeatedly issue threats, you will be asked to leave the group. Physical threats toward a group member will result in immediate expulsion from the group.
5. Listen to each other and keep harsh critical remarks to yourself. It is important to allow space for each other’s differences. However, it is intended that you let others know how they affect you personally. This way, we can learn from each other.
6. We want to start and end on time out of respect for each other.
7. Participants may not engage in intimate relationships with each other outside the group meetings.
8. Feel free to call the therapists if you have questions about the group or beyond. Often other group members have similar questions. Most questions are best addressed within the group.

Name: ___________________
Name: ___________________
Phone no.: _______________
Phone no.: _______________

**Appendix 2: Coping strategies**

Schemas are persistent and tend to become entrenched patterns in your life. The ways in which they are maintained can vary. These are the three different coping strategies:

- **Surrender**You confirm the schema by living as if it is true. For example, with a schema like *emotional deprivation*, you might unconsciously seek out partners and friends who are not able to support you emotionally.
- **Avoidance**You avoid situations where your schema might be triggered or felt. In doing so, the schema can persist because it’s never disproven. For instance, with the *emotional deprivation* schema, you might avoid entering new relationships and live in isolation. This way, the schema is not triggered but still remains.
- **Overcompensation**You are fed up with the schema and go against it head-on. You overcompensate for the schema and the emotional response it evokes. However, you do this in such a way that the schema still gets confirmed and continues to exist. For example, with *emotional deprivation*, you might become overly demanding of a partner, constantly asking for support. Eventually, your partner might leave, confirming your schema. You can recognize schema confirmation by the voice in your head saying: “See, this always happens to me.”

| **Schema** | **Surrender** | **Avoidance** | **Overcompensation** |
| --- | --- | --- | --- |
| **Abandonment/Instability** | Chooses partners who struggle with commitment and attachment. | Avoids relationships as much as possible. | Clings excessively to partner. |
| **Emotional Deprivation/Neglect** | Chooses emotionally unavailable partners. | Avoids romantic relationships. | Becomes overly demanding in contact with others, pushing them away. |
| **Mistrust/Abuse** | Chooses abusive partners. | Is distrustful and avoids vulnerability. | Uses or abuses others to meet own needs. |
| **Social Isolation/Alienation** | Focuses on differences, not similarities, with others. | Avoids groups and parties. | Tries to completely blend in, adapts excessively. |
| **Defectiveness/Shame** | Chooses rejecting relationships. Belittles self. | Does not express own emotions or opinions. | Is judgmental of others, presents as perfect. |
| **Social undesirability** | Judges own functioning in groups harshly, overemphasizes small mistakes. | Avoids social situations. | Tries very hard, mimics others, is not authentic. |
| **Failure** | Tries things half-heartedly. | Avoids challenges or tasks at work or school. | Tries extremely hard, pushes self relentlessly. |
| **Dependence** | Does not make own decisions, leaves them to others. | Avoids new tasks and challenges. | Acts overly independent, refuses to ask for help. |
| **Vulnerability to Harm/Illness** | Focuses on illnesses and disasters, consumes related media. | Avoids unsafe situations as much as possible. | Seeks out danger, shows reckless behaviour. |
| **Enmeshment/Undeveloped Self** | Focuses intensely on others, lacks personal identity and preferences. | Avoids individualistic people, stays with enmeshed others. | Becomes overly autonomous and insists on doing everything independently. |
| **Self-Sacrifice** | Always does what others want, sacrifices self. | Avoids intimate relationships, avoids situations requiring giving. | Stops doing things for others or is constantly angry at others' ingratitude. |
| **Subjugation** | Chooses controlling and dominant partners/friends. | Avoids relationships, especially those with hierarchy. | Rebels against authority or becomes passive-aggressive. |
| **Emotional inhibition** | Focuses on logic and rationality. Avoids impulsive/spontaneous behaviour. | Avoids situations that require emotional expression or spontaneity. | Becomes impulsive and uninhibited, sometimes uses alcohol or drugs to achieve this. |
| **Unrelenting standards/Criticism** | Obsessed with being perfect, spends lots of time perfecting things/self. | Avoids situations where they might be judged or evaluated. | Acts nonchalant, pretends not to care about others’ judgment. |
| **Entitlement/Grandiosity** | Exaggerates own achievements, forces opinions, insists on own way. | Avoids situations where they may not be the best. | Gives gifts and compliments to hide selfishness. |

**Appendix 3: Homework forms**

**Homework Form 1:**

**Summary:**The assignment is to write a short summary of the session. This helps you clarify for yourself what you have learned and what touched you. It can serve as a starting point for the next session.

Session number ________/ of 20
Date: ____________
Short summary of the session:
What I learned from this:

**Homework Form 2:**

**Mode Card:**

Mode 1 _______________
Mode 2 _______________
Mode 3 _______________

|  | Session  1  Load  0-10 | Session 2  Load  0-10 | Session 3  Load  0-10 | Session  4  Load  0-10 | Session  5  Load  0-10 | Session  6  Load  0-10 | Session  7  Load  0-10 | Session  8  Load  0-10 | Session  9  Load  0-10 |
| --- | --- | --- | --- | --- | --- | --- | --- | --- | --- |
| Mode 1 |  |  |  |  |  |  |  |  |  |
| Mode 2 |  |  |  |  |  |  |  |  |  |
| Mode 3 |  |  |  |  |  |  |  |  |  |

|  | Session 10  Load  0-10 | Session  11   Load  0-10 | Session  12  Load  0-10 | Session  13  Load  0-10 | Session  14  Load  0-10 | Session  15  Load  0-10 | Session  16  Load  0-10 | Session  17  Load  0-10 | Session  18  Load  0-10 |
| --- | --- | --- | --- | --- | --- | --- | --- | --- | --- |
| Mode 1 |  |  |  |  |  |  |  |  |  |
| Mode 2 |  |  |  |  |  |  |  |  |  |
| Mode 3 |  |  |  |  |  |  |  |  |  |

**Homework Form 3**

**Mode Registration**Date:
Time:

**Situation in the past week:**What happened, who was I with, where did this take place?

**Situation from the past:**How does the situation from the past week resemble a situation from the past? What was that situation? Now ask yourself the same questions about that past situation:
What happened, who was I with, where did this take place?

**Feelings:**Afraid? Yes/No Intensity of the feeling scale 1–10 _________
Angry? Yes/No Intensity of the feeling scale 1–10 _________
Sad? Yes/No Intensity of the feeling scale 1–10 _________
Ashamed? Yes/No Intensity of the feeling scale 1–10 _________
Happy? Yes/No Intensity of the feeling scale 1–10 _________

**Mode:**Do you know which mode you were in during this situation?

**Schema:**Do you know which schema was triggered?

**Schema response / coping:**How did you respond with your behavior:
Surrender: _________________________________________
Avoidance: ________________________________________
Overcompensation: _________________________________

**Conclusion:**What conclusion did you draw from the situation in the past?

What conclusion did you draw from the situation in the present?

What would you like to do differently in a similar situation in the future?

**Core need:**What underlying (core) need did you require in this situation?

1. Safety yes / no
2. Connection yes / no
3. Autonomy yes / no
4. Self-expression yes / no
5. Spontaneity and play yes / no
6. Realistic boundaries yes / no

**Homework Form 4**

**Gathering information**

*(complete this three times – once for each of your three most important schemas)*

Schema ___________________________
Thought associated with the schema ________________________________________
Credibility of the schema (0–100) __________________

**Past:**Name 2 past experiences/situations that confirm the schema

Name 2 past experiences/situations that contradict the schema

**Present:**Name 2 current experiences/situations that confirm the schema

Name 2 current experiences/situations that contradict the schema

**Present in the group:**Name 2 experiences/situations from your group therapy that confirm the schema

Name 2 experiences/situations from your group therapy that contradict the schema

Review all these experiences and situations. Reflect critically.
What is now the credibility of your schema?

Schema ______________________
Credibility: (0–100) _______________________

**Homework Form 5**

**Pros and cons analysis of your schemas and modes***(complete this three times for different schemas/modes)*

**Pros and cons of your current schema/mode**Schema/Mode ______________________________

**Advantages of this schema/mode**

**Disadvantages of this schema/mode**

**Suppose you were to change/heal the schema/mode:
Advantages of change**

**Disadvantages of change**

Review the pros and cons of this schema/mode critically.
Consider whether you want to change it. Know that it will cost you something—and also bring you something.
Are you going to change your schema/mode _______________________? YES / NO

**Homework Form 6**

**Create a pie chart for one schema**

Schema _______________________
Thought _______________________
Credibility of the schema (0–100) _______________________

**Blame and responsibility**Make a list of everyone (people, environmental factors) who are to blame or responsible for a specific situation. Make sure you list at least 4.

Assign each person a slice of the pie, and then assign a slice to yourself.

**Homework** **Form 7**

**Complete the evaluation form**

In this evaluation, you will process the results from all the Form 2 entries you have completed, into a graph.
Use a colour for each mode to show how active they have been over the past 10 weeks.

Mode 1. __________________ Colour: __________________
Mode 2. __________________ Colour: __________________
Mode 3. __________________ Colour: __________________

| 10 |  |  |  |  |  |  |  |  |  |  |
| --- | --- | --- | --- | --- | --- | --- | --- | --- | --- | --- |
| 9 |  |  |  |  |  |  |  |  |  |  |
| 8 |  |  |  |  |  |  |  |  |  |  |
| 7 |  |  |  |  |  |  |  |  |  |  |
| 6 |  |  |  |  |  |  |  |  |  |  |
| 5 |  |  |  |  |  |  |  |  |  |  |
| 4 |  |  |  |  |  |  |  |  |  |  |
| 3 |  |  |  |  |  |  |  |  |  |  |
| 2 |  |  |  |  |  |  |  |  |  |  |
| 1 |  |  |  |  |  |  |  |  |  |  |
| 0 |  |  |  |  |  |  |  |  |  |  |
|  | Week 1 | Week 2 | Week 3 | Week 4 | Week 5 | Week 6 | Week 7 | Week 8 | Week 9 | Week 10 |

Look at the graph and observe the trend. Has there been any change?

Do you see progress?

Do you notice you’re making progress in the group?

What else would you like to do to get more results?

How could the group help you grow more?

How could the therapists help you grow further?

**Homework Form 8**

Write daily in your positive diary

Day/date:

Monday : __________________                Evidence that argues against mode ____________
                                                                                      1. _____________________________   
                                                                                 2._____________________________
                                                                                      3.______________________________

Tuesday : __________________            Evidence that argues against mode ____________
                                                                                   1. _____________________________   
                                                                          2._____________________________
                                                                                    3.______________________________

Wednesday: __________________            Evidence that argues against mode ____________
                                                                                   1. _____________________________   
                                                                            2._____________________________
                                                                                      3.______________________________

Thursday : __________________            Evidence that argues against mode ____________
                                                                                   1. _____________________________   
                                                                          2._____________________________
                                                                                    3.______________________________

Friday: __________________            Evidence that argues against mode ____________
                                                                                  1. _____________________________   
                                                                            2._____________________________
                                                                                      3.______________________________

Saturday : __________________            Evidence that argues against mode ____________
                                                                                   1. _____________________________   
                                                                          2._____________________________
                                                                                    3.______________________________

Sunday: __________________            Evidence that argues against mode ____________
                                                                                   1. _____________________________   
                                                                          2._____________________________
                                                                                    3._____________________________
