# Supplementary material for: Feasibility of online group schema therapy: A preliminary study with therapists in training for future application in borderline personality disorder
Source: Internet Interv. 2025 Dec 5;43:100897. doi: 10.1016/j.invent.2025.100897 (PMC12768866; doi:10.1016/j.invent.2025.100897)
Supplement: Annex 4 — Document transitional objects. [file mmc4.docx]

**Annex 4- Document transitional objects**

**Dear participant of the online schema therapy group,**

You are receiving a number of items that we will occasionally use during the upcoming sessions for various exercises. Please make sure to keep these items within reach during the sessions.

The items are:

1. **Bubble blower** 🡪 used for “happy child” exercises. These exercises can help break the tension, change the pace, or end a session pleasantly. For example, you could have a contest to see who can blow the biggest or the most bubbles on screen.
   *(Anne, can you check if there are more exercises that use the bubble blower?)*
2. **Balloons** 🡪 used in various exercises related to anger or sadness.
   (See the “angry child” session > anger exercise, note this one here. They can also be used in a similar way for sadness.)
3. **Transparent pebble** 🡪 represents the group, the connection through glass. Keep it with you or bring it out when working on homework.
4. **Clay** 🡪 this exercise is part of the “Demanding Parent” session (see that session for details).
5. **Ball of yarn** 🡪 for the vulnerable child to hold when needed. It symbolizes connection and safety.
6. **Seeds** 🡪 more explanation will follow in the group. The idea is that everyone plants these seeds at the beginning of the group and, with good care, enough water, and warmth, lets them grow. Keep each other updated on the progress. Enjoy the watercress once it has grown.
7. **Candle** 🡪 more explanation will follow in the group. You can light this candle at the beginning of each session and also when working on homework assignments.
8. **Candy** 🡪 hopefully, you’ve already enjoyed it! These are for you.
9. **Hairband** 🡪 symbolizes the group and connectedness. You can wear it daily to remind yourself of the group, the modes, and the exercises.
10. **Party horn** 🡪 used for “happy child” exercises, usually at the end of the session to close on a positive note.
