# Supplementary material for: Feasibility of online group schema therapy: A preliminary study with therapists in training for future application in borderline personality disorder
Source: Internet Interv. 2025 Dec 5;43:100897. doi: 10.1016/j.invent.2025.100897 (PMC12768866; doi:10.1016/j.invent.2025.100897)
Supplement: Annex 5 — CTS-1 Questionnaire. [file mmc5.docx]

**Band met de therapeut schaal (CTS)**

INSTRUCTIE

Deze vragenlijst gaat over uw relatie met uw groepstherapeut. Bij twee groepstherapeuten vult u de vragenlijst in over de voor u *belangrijkste* therapeut.

Beantwoord verder eerst de volgende vraag door het *aankruisen van één van de twee rondjes*.

**De therapeut over wie ik de onderstaande vragenlijst invul:**

**O is alleen als groepstherapeut verbonden aan mijn groep**

**O is behalve mijn groepstherapeut ook mijn individuele therapeut**

Richt u zich bij de volgende uitspraken op uw indrukken van de door u gekozen therapeut.

Omcirkel de ‘x’ die volgens u **van toepassing** is.

| **Helemaal niet van toepassing** | |  |  |  |  | **Helemaal van toepassing** |
| --- | --- | --- | --- | --- | --- | --- |
| Ik vertrouw hem/haar........................................................... | x | x | x | x | x | x |
| Ik wou dat hij/zij meer zijn/haar gevoelens uitte................. | x | x | x | x | x | x |
| Ik zou wel vriendschap met hem/haar willen sluiten  buiten de groep..................................................................... | x | x | x | x | x | x |
| Ik respecteer hem/haar......................................................... | x | x | x | x | x | x |
| Ik wou dat hij/zij meer aandacht aan me schonk................. | x | x | x | x | x | x |
| Ik ken hem/haar.................................................................... | x | x | x | x | x | x |
| Ik mag hem/haar graag......................................................... | x | x | x | x | x | x |
| Ik wou dat hij/zij actiever was............................................. | x | x | x | x | x | x |
| Ik lijk op hem/haar (ik deel sommige dingen met  hem/haar) ............................................................................. | x | x | x | x | x | x |

**Relationship with the Therapist Scale (CTS)**

INSTRUCTION

This questionnaire concerns your relationship with your grouptherapist. In the case of two grouptherapists, fill this questionnaire in regarding your relationship with the grouptherapist that is most important to you. Answer the following question by crossing off óne of the two circles below.

**The therapist about which I’m taking the questionnaire below:**

**O is just connected to my group as grouptherapist**

**O is also my individual therapist outside of being my grouptherapist**

For the following statements, focus on your impressions of your chosen therapist.

Circle the "x" that you think applies.

| **Completely not applicable** | |  |  |  |  | **Completely applicable** |
| --- | --- | --- | --- | --- | --- | --- |
| I trust him/her (the therapist)............................................... | x | x | x | x | x | x |
| I wish he/she was more open about his/her feelings............ | x | x | x | x | x | x |
| I would like to make friends with him/her outside the group | x | x | x | x | x | x |
| I respect him/her……........................................................... | x | x | x | x | x | x |
| I wish he/she would pay more attention to me…................. | x | x | x | x | x | x |
| I know him/her ..................................................................... | x | x | x | x | x | x |
| I like him/her ………........................................................... | x | x | x | x | x | x |
| I wish he/she would be more active ..................................... | x | x | x | x | x | x |
| I am like him/her (I share some interests with him/her)........ | x | x | x | x | x | x |
